# Enhancement of Antimicrobial Function by L/D-Lysine Substitution on a Novel Broad-Spectrum Antimicrobial Peptide, Phylloseptin-TO2: A Structure-Related Activity Research Study

**DOI:** 10.3390/pharmaceutics16081098

**Published:** 2024-08-21

**Authors:** Weining Yin, Junting Yao, Xuwei Leng, Chengbang Ma, Xiaoling Chen, Yangyang Jiang, Tao Wang, Tianbao Chen, Chris Shaw, Mei Zhou, Lei Wang

**Affiliations:** Natural Drug Discovery Group, School of Pharmacy, Queen’s University Belfast, Belfast BT9 7BL, UK; wyin02@qub.ac.uk (W.Y.); jyao05@qub.ac.uk (J.Y.); xleng01@qub.ac.uk (X.L.); c.ma@qub.ac.uk (C.M.); yangyang.jiang@qub.ac.uk (Y.J.); t.wang@qub.ac.uk (T.W.); t.chen@qub.ac.uk (T.C.); chris.shaw@qub.ac.uk (C.S.); m.zhou@qub.ac.uk (M.Z.); l.wang@qub.ac.uk (L.W.)

**Keywords:** antimicrobial peptide, phylloseptin, D-lysine substitution, membrane interaction, anti-cancer activity

## Abstract

Antibiotic resistance poses a serious threat to public health globally, reducing the effectiveness of conventional antibiotics in treating bacterial infections. ESKAPE pathogens are a group of highly transmissible bacteria that mainly contribute to the spread of antibiotic resistance and cause significant morbidity and mortality in humans. Phylloseptins, a class of antimicrobial peptides (AMPs) derived from *Phyllomedusidae* frogs, have been proven to have antimicrobial activity via membrane interaction. However, their relatively high cytotoxicity and low stability limit the clinical development of these AMPs. This project aims to study the antimicrobial activity and mechanisms of a phylloseptin-like peptide, phylloseptin-TO2 (PSTO2), following rational amino acid modification. Here, PSTO2 (FLSLIPHAISAVSALAKHL-NH_2_), identified from the skin secretion of *Phyllomedusa tomopterna*, was used as the template for modification to enhance antimicrobial activity. Adding positive charges to PSTO2 through substitution with L-lysines enhanced the interaction of the peptides with cell membranes and improved their antimicrobial efficacy. The analogues SRD7 and SR2D10, which incorporated D-lysines, demonstrated significant antimicrobial effects against *Staphylococcus aureus* and methicillin-resistant *Staphylococcus aureus* (MRSA) while also showing reduced haemolytic activity and cytotoxicity, resulting in a higher therapeutic index. Additionally, SRD7, modified with D-lysines, exhibited notable anti-proliferative properties against human lung cancer cell lines, including H838 and H460. This study thus provides a potential development model for new antibacterial and anti-cancer drugs combating antibiotic resistance.

## 1. Introduction

Infections caused by antimicrobial-resistant pathogens majorly impact public health [1,2]. Conventional antibiotics, a group of chemical agents produced by bacteria and fungi, are widely used to combat bacterial infections by killing pathogens or preventing their growth [3]. Antibiotics target pathogens through various mechanisms, including disrupting the synthesis of cell walls, genetic materials and essential proteins, alternating cell membranes, and inhibiting the synthesis of essential metabolites [4]. However, the exposure of bacteria to antibiotics has led to the development and selection of antibiotic-resistant strains, weakening the effectiveness of traditional treatments [5]. ESKAPE is a group of six highly virulent and antibiotic-resistant bacteria, and their biofilms can cause chronic infections [6]. These antibiotic-resistant bacteria pose serious threats to human health and the healthcare system because they are difficult to treat, leading to prolonged infections and higher mortality rates [7]. Infections can also lead to inflammation, and chronic inflammation can cause DNA damage, which promotes cancer growth through genotoxicity, increased cell proliferation, and cancer cell invasion [8]. Thus, developing novel antimicrobial alternatives is urgently needed due to the increased difficulty in treating infections.

Antimicrobial peptides (AMPs) are generally 12–50 amino acids in length and contain hydrophobic and cationic amino acids. AMPs originating from amphibian skin secretion are proven to have inhibitory and bactericidal effects against multiple microorganisms [9,10,11]. As essential parts of the amphibian immune system, AMPs are produced from their granule and mucus glands and secreted via a holocrine pathway [12]. Phylloseptin, a peptide found in frogs of the *Phyllomedusa* genus, has gained attention as a promising antibacterial agent since its first isolation from the tiger leg monkey frog, *Phyllomedusa hypochondrialis*, in 2004. It has demonstrated strong inhibitory effects against various bacteria and negligible haemolytic activity [13]. Members of the phylloseptin family share similar structural characteristics, including a consistent, highly conserved N-terminal sequence (FLSLIP-) and C-terminal amidation. Functionally, phylloseptins are active against Gram-negative and Gram-positive bacteria and have anti-cancer properties [14,15,16]. However, the limitations of AMP therapy have also been of concern [17]. Many AMPs are sensitive to environmental factors, such as pH, temperature, and proteases, which can limit their efficacy and shelf life and affect future development and clinical applications [18]. Therefore, researchers have been trying to modify naturally isolated AMPs artificially to enhance their biological activity and therapeutic effect while minimising their toxicity.

In this study, a novel AMP of the phylloseptin family, named phylloseptin-TO2 (PSTO2), was identified from the skin secretion of the tiger-striped frog *Phyllomedusa tomopterna*. The peptide was subject to bioinformatics and bioactivity analyses after synthesising by solid-phase peptide synthesis (SPPS) and purifying by reverse-phase high-performance liquid chromatography (RP-HPLC). Then, a series of analogues were designed by substituting critical amino acid sites. The anti-microorganism function, anti-cancer ability, cytotoxicity, and potential acting mechanism were evaluated in an attempt to find a better candidate with potent bactericidal activity, selectivity, stability, and low toxicity. Moreover, studying the relationship between peptide structure and bioactivity could contribute to further artificially modified peptide engineering.

## 2. Materials and Methods

### 2.1. Collection of Skin Secretion in Phyllomedusa tomopterna

*Phyllomedusa tomopterna* (n = 4) were bought from a commercial source in the United States and maintained in Queen’s University Belfast Animal Facility. The skin secretion from the frogs was non-invasively collected by gentle electrical stimulation (5V; 3 ms pulses) for 30 s on the dorsal. The harvest of skin secretion was performed by Dr Mei Zhou under the UK Animal (Scientific Procedures) Act 1986 and approved with a project license on 1 March 2011 (code: PPL 2694), issued by the Department of Health, Social Services, and Public Safety, Northern Ireland, UK.

### 2.2. Identification of Precursor-Encoding cDNA of PSTO2

Five mg of lyophilised skin secretion of *Phyllomedusa tomopterna* was dissolved in 1 mL of lysis/binding buffer (Dynal Biotech, Merseyside, UK) in an autoclave micro-centrifuge tube. A Dynabeads^®^ mRNA Direct^TM^ kit (Dynal Biotech, Merseyside, UK) was used as an effective method to extract the poly-A tail of mRNAs. The resultant mRNAs were utilised to construct a cDNA library using a SMART RACE cDNA Amplification Kit (BD Biosciences Clontech, Basingstoke, UK). Then, the entire length of the precursor cDNAs was obtained from the amplification procedure with the 3′-RACE PCR process, along with a degenerate sense primer (5′-ACTTTCYGAWTTRYAAGMCCAAABATG-3′), which was designed based on the highly conserved domain of the 5′-untranslated region of previously characterised AMP precursor-encoding cDNAs from *Phyllomedusa* species. The products were verified by gel electrophoresis, and the target DNA products were purified with a MiBind DNA Mini Column in a Cycle Pure Kit (Omega Bio-Tek, Norcross, GA, USA). After that, the DNA products were ligated with the pGEM^®^ -T Easy Vector system (Promega, Madison, WI, USA) to yield recombinant DNAs, which were expressed and amplified by the transformation process with *Escherichia coli* (*E. coli*) JM109. The amplified DNA products were selected with a white and blue screening, and the sequence was identified with an ABI 3100 automatic capillary sequencer (Applied Biosystems, Foster City, CA, USA). The nucleotide sequence was translated with Expasy (https://web.expasy.org/translate/, accessed on 8 March 2023) and analysed with the online server NCBI-BLAST (https://blast.ncbi.nlm.nih.gov/Blast.cgi, accessed on 5 April 2023).

### 2.3. Peptide Design and Physicochemical Characteristics Prediction

The secondary structure of PSTO2 and its analogues were predicted with PEP-FOLD 3 (https://bioserv.rpbs.univ-paris-diderot.fr/services/PEP-FOLD3/, accessed on 5 May 2024), and the properties were analysed by Heliquest (https://heliquest.ipmc.cnrs.fr/, accessed on 5 May 2024) to design the analogues of PSTO2. After importing the complete sequence into the website, the composition of amino acids, physicochemical characteristics, and helix structure were demonstrated. Different amino acids are distinguished according to their properties and represented by different colours. Based on these analyses, we replaced some uncharged amino acids with lysines to obtain analogues 7K, 18K, SR, and SR2, each with higher total positive charges. Additionally, several D-lysine substituted analogues, namely, SRD7, SR2D10, D1, and D2, were designed to examine the effect of the conformational differences.

### 2.4. Peptide Synthesis and Identification of PSTO2 and Its Analogues

To acquire sufficient peptide product of PSTO2 and its analogues, a Tribute^®^ peptide synthesiser (Protein Technologies, Tucson, AZ, USA) was used along with different fluorene methoxycarbonyl amino acids (Fmoc amino acids). Then, MBHA resin (Millipore Sigma, Burlington, MA, USA) was used as the carrier, and the amino acids required by the sequence were linked and synthesised. The 2-(1H-benzotriazole-1-yl)-1,1,3,3-tetramethyluronium hexafluorophosphate (HBTU) together with 11% 4-methylmorpholine in dimethylformamide (DMF) were used to catalyse the carboxyl. The finished peptide was cleaved by the mixture of 94% trifluoroacetic acid (TFA), 2% deionised water (dd H_2_O), 2% thiamazole, and 2% 1,2-ethanedithiol in the condition of stirring at room temperature in a fume hood. Afterwards, the crude peptide was precipitated and washed with diethyl ether (DEE). Then, the peptide was dissolved with 5 mL of HPLC solution A (dd H_2_O/TFA = 0.05/99.95, *v*/*v*) and 25 mL of HPLC solution B (TFA/H_2_O/acetonitrile = 0.05/19.95/80.00, *v*/*v*/*v*), which was followed by lyophilisation.

RP-HPLC (Waters^®^, Milford, MA, USA) was used to purify the crude peptides. The whole system consisted of an infusion pump (Adept CECIL CE4100), a degasser (Adept CECIL CE4020), an ultraviolet detector (Adept CECIL CE4200), and a column (Jupiter 5u C18 300A 250 × 21.20 mm) constructed together with Power Stream software (version 3.1). The concentration of the peptide sample was 1 mg/mL, which was dissolved in HPLC solution A (dd H_2_O/TFA = 0.05/99.95, *v*/*v*), and the volume of each injection was 5 mL. The column of the HPLC machine was washed with HPLC solution B and balanced with HPLC solution A. The wavelength was set up to 214 nm, and the flow rate was 5 mL/min with the gradient from 100% HPLC solution A to 100% HPLC solution B. All fragments with a response value greater than 0.5 were collected separately.

Matrix-assisted laser desorption ionisation time-of-flight (MALDI-TOF) (Voyager DE, PerSpective Biosystems, Framingham, MA, USA) mass spectrometry was used to determine the mass-to-charge ratio (*m*/*z*). To prepare the matrix used in this process, 1 mg of α-cyano-4-hydroxycinnamic acid (CHCA) was dissolved in 100 μL of acetonitrile/dd H_2_O (70/29.98, *v*/*v*) with 0.02% TFA at 10 mg/mL. Two µL of each HPLC fraction was transferred to a spot on a MALDI-TOF plate and dried at room temperature. Then, the spots were covered with 1 μL of the CHCA matrix, and the plate was transferred into the system to record the mass spectra. The fraction with matching predicted *m*/*z* was selected for further bioactivity assays.

### 2.5. Secondary Structure Detection by Circular Dichroism (CD) Spectroscopy

CD spectroscopy was used to confirm the secondary structure of these peptides. This technique is underpinned by the different absorption of circularly polarised light by substances with various structural features, resulting in different CD spectra. For example, a peptide or protein containing an α-helical structure has negative bands at 222 nm and 208 nm and positive bands at 193 nm.

A JASCO J-815 CD spectrometer (JASCO Inc., Tokyo, Japan) was used and set up as described in a previous publication [19]. Peptide samples at a concentration of 100 μM were, respectively, dissolved in 10 mM of ammonium acetate buffer (NH_4_Ac) (aqueous environment) and 50% TFE/NH_4_Ac *v*/*v*) (membrane–mimetic environment). CD spectra data were displayed as the mean residue ellipticity (θ) in deg·cm^2^·dmol^−1^. The collected data were analysed using the online web service K2D3 (http://cbdm-01.zdv.uni-mainz.de/~andrade/k2d3/, accessed on 13 March 2024).

### 2.6. Minimum Inhibitory Concentration (MIC) and Minimum Bactericidal Concentration (MBC) Screening Assays

Seven microorganism strains were used to preliminarily evaluate the antimicrobial activity of PSTO2 and its analogues, including *Staphylococcus aureus* (*S. aureus*) (ATCC^®^ 6538), methicillin-resistant *Staphylococcus aureus* (MRSA) (NCTC^®^ 12493), *Enterococcus faecalis* (*E. faecalis*) (NCTC^®^ 12697), *E. coli* (ATCC^®^ 8739), *Klebsiella pneumonia* (*K. pneumonia*) (ATCC^®^ 43816), *Pseudomonas aeruginosa* (*P. aeruginosa*) (ATCC^®^ CRM 9027), *Acinetobacter baumanni* (*A. baumanni*) (BAA^®^ 747), and *Candida albicans* (*C. albicans*) (ATCC^®^ 10231). In this study, the strains were cultured in Mueller–Hinton broth (MHB), nutrient broth (NB), and yeast–peptone–dextrose broth (YPD-B) correspondingly at 37 °C, with shaking at 120 rpm. Each peptide was dissolved from 128 μM to 1 μM and mixed with the bacterium at 5 × 10^5^ CFU/mL. In this process, bacteria with culture medium were used as the growth control group; PBS was set up as the vehicle control group; sterile culture medium was used as the blank control group; and Norfloxacin at a concentration of 2 mg/mL and amphotericin B at a concentration of 1 mg/mL were set up as the positive control group for bacteria and fungi, respectively.

After being incubated for 24 h, a Synergy HT plate reader (Bio-Tek, Shoreline, WA, USA) was used to detect the absorbance of each well in the 96-well plate at λ = 550 nm. The concentration at which no visible turbidity could be recorded was the MIC value. Beginning from the MIC value, 10 µL of solution in each clear well was dropped on the sterile agar plates. The plate was checked the next day for colony formation, and the lowest concentration at which no colony growing was recorded as an MBC value.

### 2.7. Minimum Biofilm Inhibitory Concentration (MBIC) and Minimum Biofilm Eradication Concentration (MBEC) Evaluation

To test the anti-biofilm ability of PSTO2 and its analogues, three types of microorganisms (*S. aureus* ATCC^®^ 6538, *E. coli* ATCC^®^ 8739, and MRSA NCTC^®^ 12493) were chosen. Tryptic soy broth (TSB) and Lysogeny broth (LB) were used to culture microorganisms. The peptide was dissolved in an autoclaved PBS solution and diluted into concentrations ranging from 51,200 µM to 100 µM. One µL of peptide solution of each concentration was added into a 96-well plate with 100 µL bacterium at 5 × 10^5^ CFU/mL. The plate was incubated at 37 °C for 24 h, with shaking at 200 rpm. After overnight incubation, the planktonic bacteria were washed up. Then, 100 µL of methanol was used to fix the biofilms onto the bottom, and 100 µL of 0.1% crystal violet solution (Sigma-Aldrich, Gillingham, UK) was added to each well to stain the biofilms. After drying, 160 µL of acetic acid (30% in deionised water, Sigma-Aldrich, UK) was added to each well to dissolve the shaded parts. The absorbance was detected with the Synergy HT plate reader at λ = 595 nm.

For the MBEC assay, 100 µL of the bacterium of 5 × 10^5^ CFU/mL was transferred into an empty 96-well plate, and the plate was incubated at 37 °C for 24 h to obtain the biofilms primarily. Then, the peptide solution from 512 µM to 1 µM was transferred into a corresponding well, followed by 24 h incubation at 37 °C, with shaking at 200 rpm. After this, the plate was handled using the identical method to the plate in the MBIC assay, and the absorbance of each well was detected with the Synergy HT plate reader at λ = 595 nm. The minimum concentration at which the peptide can eradicate the existing biofilm was recorded as the MBEC value.

### 2.8. Time-Killing Kinetic Assays

To examine the antimicrobial kinetics of PSTO2 and its analogues, a time-killing assay was conducted with three types of bacteria (*S. aureus* ATCC^®^ 6538, *E. coli* ATCC^®^ 8739, and MRSA NCTC^®^ 12493). Each strain was cultured with peptides at 1× MIC, 2× MIC, and 4× MIC in a 96-well plate. Ten µL of the content of each group was diluted in 90 µL of sterile PBS in 10-fold, which was repeated 6 times, and transferred onto a Nutrient Agar plate at different time points (0, 5, 10, 20, 30, 60, 90, 120, and 180 min) for live cell counting. The colonies were quantified after 24 h incubation at 37 °C.

### 2.9. Salt and Serum Sensitivity Detection

To evaluate the antimicrobial efficacy of PSTO2 and its analogues under salt and serum conditions, seven types of salt ions and 10% fetal bovine serum (FBS) were prepared to physiological concentrations (NaCl, 150 mM; KCl, 4.5 mM; CaCl_2,_ 2.5 mM; NH_4_Cl, 6 μM; ZnC_l2_, 8 μM; MgCl_2_, 1 mM; and FeCl_3_, 4 μM) in TSB/NB medium. The chosen microorganisms (*S. aureus* ATCC^®^ 6538 and MRSA NCTC^®^ 12493) were cultured to 5 × 10^5^ CFU/mL in a prepared medium and mixed with different concentrations of salt solution and FBS. Then, the MIC assay of each peptide was conducted, as previously described in Section 2.6.

### 2.10. Sytox Green Permeability Assays

Sytox^TM^ Green Nucleic Acid Stain (Life Technologies, Paisley, UK) was used to detect the permeability of the bacteria cell membranes, which were affected by PSTO2 and its analogues. Different strains (*S. aureus* ATCC^®^ 6538 and MRSA NCTC^®^ 12493) were incubated to the log phase and centrifugally washed with 30 mL of 5% TSB/0.85% NaCl twice at 1000× *g*. Then, 50 μL of bacteria, 40 μL of peptide solution, and 10 μL of 1% Sytox Green dye (dissolved in TSB/0.85% NaCl buffer) were mixed and incubated for 2 h. For this assay, bacteria with 5% TSB/0.85% NaCl were used as the growth control group, and sterile PBS was set up as the vehicle control group. The plate was analysed with a Synergy HT plate reader (BioTek, Shoreline, WA, USA), with excitation at 485 nm and emission at 528 nm.

### 2.11. Outer Membrane Permeability Assays

*N*-Phenyl-1-naphthylamine (NPN), a hydrophobic fluorescent probe, can bind to the hydrophobic tail of phospholipids and becomes fluorescent when outer membrane rupture occurs. The permeability of the outer membrane was evaluated with *E. coli* ATCC^®^ 8739 through an NPN-intake assay, as previously described [20]. The bacteria were inoculated in TSB medium and incubated in a shaking incubator at 200 rpm at 37 °C overnight. The overnight inoculum (500 µL) was sub-cultured in 25 mL of TSB medium for another 2 h at 37 °C with shaking at 200 rpm to obtain sufficient bacteria. Next, the cells were pelleted at 3000× *g* for 10 min and washed three times through resuspension in 5 mM of HEPES. The cells were diluted with 5 mM of HEPES (with 5 mM of glucose) to a final OD_600_ = 0.5, which corresponds to the concentration of 1 × 10^8^ CFU/mL. Then, the bacteria suspension was diluted in 5 mM of HEPES and 5 mM of glucose buffer to 1 × 10^7^ CFU/mL, and 100 μL of the diluted bacterial suspension was mixed with 50 μL of peptide sample and 50 μL of 40 μM NPN in a black 96-well plate. The fluorescence intensity of NPN was determined using a Synergy HT plate reader (BioTek, Shoreline, WA, USA) with excitation at 360 nm and emission at 460 nm for 2 h with 90 s intervals.

### 2.12. Membrane Potential Assays

The change in the inner membrane potential of *E. coli* (ATCC 8739) affected by PSTO2 and its analogues was verified with the fluorometric variation in the voltage-sensitive 3,3′-Dipropylthiadicarbocyanine Iodide (DiSC_3_-5) (Sigma, St. Louis, MO, USA). The bacteria were cultured in LB medium overnight and sub-cultured for another 2 h at a speed of 120 rpm and a 37 °C temperature. Then, the bacteria were washed with 5 mM of HEPES (with 20 mM of glucose) and centrifuged at 3000× *g* for 10 min to separate the medium. This step was repeated 3 times. Following this, the cell pellets were diluted with 5 mM of HEPES (with 20 mM of glucose) to a final OD_600_ = 0.5, and 10 mL of diluent was mixed with 200 μL of 20 μM DiSC_3_-5 in a 50 mL tube at room temperature for 1 h. Subsequently, ten microliters of different concentrations of peptide were added into a black 96-well plate together with 90 μL of bacteria suspension, and the plate was analysed with a Synergy HT plate reader (BioTek, Shoreline, WA, USA) for 30 min at 1 min intervals, with the excitation wavelength set at λ = 485 nm and the emission wavelength set at λ = 645 nm.

### 2.13. In Vivo Antimicrobial Activity of Peptides

The antimicrobial effect of PSTO2 and its selected analogues against MRSA in vivo was tested with *Galleria mellonella* (Live Food, Axbridge, UK). Ten waxworms (250 ± 20 mg) were selected for each treatment group, and 10 μL of bacteria suspension (5 × 10^7^ CFU/mL) was injected to infect each worm. After 1 h of infection, each worm was administered 10 µL of 8 mg/kg and 16 mg/kg of PSTO2, SRD7, and SR2D10. Vancomycin was set up as a positive control. All the waxworms were incubated at 24 °C and observed every 24 h for five days. To score for worm survival after peptide treatment, worms that changed colour from golden to dark brown/black and lacked movement when manipulated with a pipette tip were recorded as dead.

### 2.14. Anti-Proliferative Effect Evaluation

The percentage of cancer cell survival after being treated with peptides was assessed with 3-(4,5-dimethylthiazol-2-yl-)-2,5-diphenyltetrazolium bromide (MTT). The principle is the reduction process of the yellow tetrazolium to DMSO-soluble formazan crystals caused by the metabolic capability of living cells. All the cancerous and normal cells were obtained from the American Type Culture Collection (Rockville, VA, USA). Human lung cancer NCI-H838 and NCI-H460 were cultured in Roswell Park Memorial Institute (RPMI)-1640 medium (Gibco, Paisley, UK) and mixed with 10% (*v*/*v*) fetal bovine serum (FBS) (Gibco, Campinas, Brazil) and 1% penicillin–streptomycin (Gibco, Grand Island, NY, USA) 89/10/1 *v*/*v*/*v*). U251MG and MCF-7 were cultured in Dulbecco’s Modified Eagle medium (DMEM) (Gibco, Paisley, UK) and mixed with 10% FBS and 1% penicillin–streptomycin. HCT 116 was cultured in McCoy’s 5A (modified) medium (Gibco, Paisley, UK) and mixed with 10% FBS and 1% penicillin–streptomycin. MRC-5 and HaCat were cultured in Minimum Essential Medium (MEM) (Gibco, Paisley, UK) and mixed with 10% FBS and 1% penicillin–streptomycin. Cells were cultured and seeded in a 96-well plate at a density of 8000 cells/well with the corresponding medium and incubated for 24 h at 37 °C with 5% CO_2_. After 24 h of incubation, the cells were starved with a serum-free medium for 4 h at 37 °C. Then, the cells were incubated with different concentrations of peptide solution in a serum-free medium from 1 nM to 100 µM at 37 °C for 24 h. Meanwhile, triton X-100 (0.1%) (Sigma-Aldrich, St. Louis, MO, USA) and PBS were chosen as positive and negative controls. After 10 µL of MTT (5 mg/mL) was added to each well away from light and incubated at 37 °C for 2 h, the formazan generated by mitochondria was dissolved with DMSO. The absorbance was detected at a wavelength of 570 nm by a Synergy HT plate reader (Bio-Tek, Shoreline, WA, USA).

### 2.15. Trypan Blue Exclusion Assays

Trypan blue was used to detect the killing kinetics against cancer cells of PSTO2 and its selected analogues. NCI-H838 cells were cultured in RPMI-1640 complete medium as the anti-proliferation assay, as previously mentioned in Section 2.14, and were seeded into a 12-well plate with 2 × 10^5^ cells per well. The seeded plates were incubated at 37 °C with 5% CO_2_ overnight. Subsequently, 500 µL of peptide solution prepared in a serum-free medium was added to each well to achieve the final concentration of 25 µM, 10 µM, and 5 µM after starvation. At each time point (2 h, 6 h, and 24 h), entire cells were digested and collected separately. Ten µL of cell suspension was mixed with the equivalent volume of trypan blue (0.4%, Gibco, Grand Island, NY, USA) for cell counting with a haemocytometer. The percentage of survival rate was calculated with the ratio of living cells in the peptide group and negative control group.

### 2.16. Haemolysis Activity Evaluation

To assess the haemolytic activity of PSTO2 and its analogues, 2% fresh defibrinated horse red blood cells (TCS Biosciences Ltd., Buckingham, UK) were acquired after being washed and resuspended in sterile PBS. Each peptide was dissolved in PBS to prepare solutions with concentrations ranging from 2 µM to 1024 µM. Subsequently, 100 µL of each peptide solution was mixed with 100 µL of the red blood cell suspension. The red blood cells treated with 1% triton X-100 and sterile PBS in equivalent volumes were set up as the positive and negative control groups, respectively. Then, all the samples were incubated at 37 °C for 2 h. Thereafter, the samples were centrifuged at 900× *g* for 10 min. Finally, 100 µL of supernatant of each sample was transferred into a 96-well plate, and the absorbance was recorded by a Synergy HT plate reader (Bio-Tek, Shoreline, WA, USA) at a wavelength of 570. The following equation was used to calculate haemolysis activity:

Haemolysis activity = (Sample absorbance − A_b_)/(A_p_ − A_b_) × 100%

where A_b_ refers to the mean value of absorbance of the negative control (incubated with PBS only), and A_p_ refers to the mean absorbance value of the positive control (incubated with triton X-100).

### 2.17. Statistical Analysis

Nine replications of data were acquired after 3 individual tests in all the experiments. All the data were analysed by the GraphPad Prism 9 software (GraphPad, San Diego, CA, USA). The error bars in all the graphs were generated based on the standard error of the mean (SEM), and the *p*-value was analysed with one-way ANOVA tests. 

## 3. Results

### 3.1. Shotgun Cloning of PSTO2 Precursor Encoding cDNA from Phyllomedusa tomopterna Frog Skin Secretion

**Figure 1:**
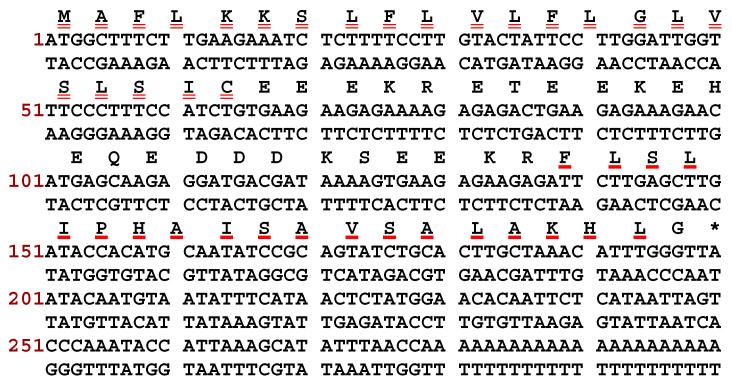
Nucleotide sequence of cDNA encoding precursor and corresponding amino acid residues of PSTO2. The signal peptide is labelled with double underlines, the amino acid composition of PSTO2 is marked with a bold line, and the termination codon is indicated by an asterisk.

**Figure 2:**
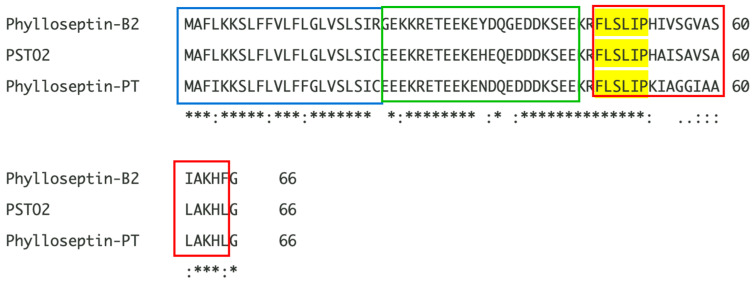
Alignment of the peptide encoding precursor of PSTO2 and two highly similar mature peptides (phylloseptin-B2 and phylloseptin-PT) sequences of the phylloseptin family. The signal peptide sequences are highlighted in the blue box, the acid spacer regions are highlighted in the green box, and the mature peptides are highlighted in the red box. The conserved motif ‘FLSLIP’ in phylloseptins is highlighted in yellow. The asterisks (*) represent identical amino acid sequences, the colons (:) indicate conservation between groups with very similar properties, and the full stops indicate conservation between groups with weakly similar properties.

The PSTO2 encoding precursor was identified from the skin secretion of *Phyllomedusa tomopterna* using the ‘shotgun’ cloning method (Figure 1). The translated open-reading frame includes 66 amino acid residues and can be divided into different parts. The signal peptide domain, which was double underlined, was composed of 22 amino acid residues and started with methionine (M) at the N-terminal and ended with cysteine (C) at the C-terminus. Following this, a hydrophilic domain with 24 amino acid residues that formed the acidic spacer region ended with a representative cleavage site -Lys-Arg- (-K-R-). A glycine residue at the end of the sequence was regarded as a traditional amide donor; thus, the mature peptide sequence was decided to be FLSLIPHAISAVSALAKHL-NH_2_, labelled with the bold red underline. According to the NCI-BLAST analysis results, this mature sequence owns the consistently conserved motif ‘FLSLIP’ with others of the phylloseptin subfamily, so this novel peptide belongs to phylloseptin and is named PSTO2 (Figure 2).

### 3.2. Rational Design and Property Analysis

**Figure 3:**
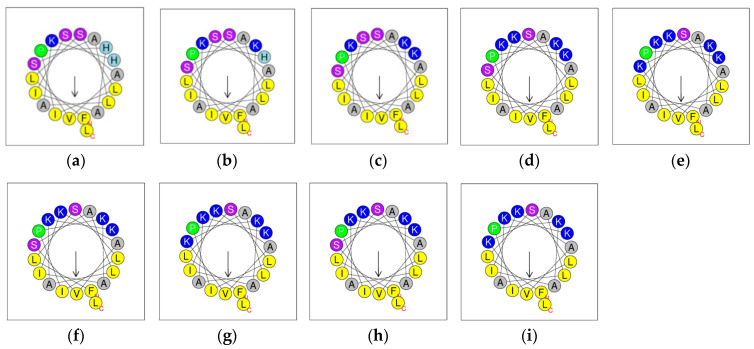
Predicted helical wheels of PSTO2 and its analogues (**a**–**i**). Each arrow indicates the hydrophobic face of the peptide. Different colours represent the various properties of the amino acids (yellow: hydrophobic residues; grey: non-polar residue; other colours: corresponding amino acids).

PSTO2 was utilised as a template, and eight analogues were designed by amino acid substitution based on the helical wheel prediction graphs, with the hydrophobic face of each peptide highlighted in yellow (Figure 3). The helical wheel projections visually represent the distribution of hydrophobic and hydrophilic residues around the helix. This visualisation is crucial for understanding the spatial arrangement and ensuring that lysine substitutions are made on the hydrophilic face without disrupting the hydrophobic face, which is essential for maintaining peptide–membrane interactions. Based on these predictions, the influence of positive charges was evaluated, and the cation summation of 7K, 18K, SR, and SR2 was improved by replacing histamine and serine with lysine in different positions, increasing the peptide charge from +1 (PSTO2) to +5 (SR2). The sequences, net charge, and hydrophobicity of these peptides are detailed in Table 1. Additionally, SR and SR2 were used as templates, and the lysines in different positions were substituted with D-type lysine enantiomers, yielding the analogues of SRD7, SR2D10, D1, and D2.

### 3.3. Peptide Synthesis and Identification

Each peptide was synthesised on a Tribute^®^ peptide synthesiser at a 0.3 mmol scale. After synthesis, PSTO2 and its analogues were purified using RP-HPLC, and the purity of the peptides (>95%) was checked by analytical HPLC, as shown in Figure S1. The mass-to-charge ratio (*m*/*z*) of each peptide was determined by MALDI-TOF spectrometry, and the spectroscopic graphs are demonstrated in Figure S2.

### 3.4. Secondary Structure Analysis of PSTO2 and Its Analogues

**Figure 4:**
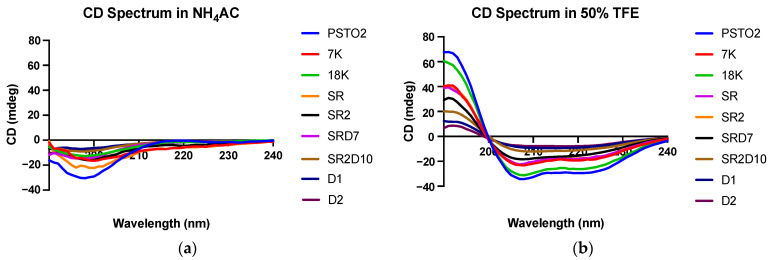
CD spectra of PSTO2 and its analogues in (**a**) 50% TFE/NH_4_Ac solution and (**b**) 10 mM of NH_4_Ac. CD spectra data are displayed as measured ellipticity in mdeg units.

The secondary structure of PSTO2 and its analogues was confirmed by their CD spectra (Figure 4). PSTO2 and its analogues exhibited a random coil structure in the aqueous environment, characterised by a significant negative peak at λ = 198 nm in the spectra. Meanwhile, these peptides showed an alpha-helical conformation in the membrane environment simulated by 50% TFE/10 mM NH_4_Ac, with double minima at approximately 208 nm and 222 nm, which is a typical signature of alpha-helical structures. The helical percentage of each peptide was calculated by K2D3 (Table 2) (http://cbdm-01.zdv.uni-mainz.de/~andrade/k2d3/, accessed on 5 May 2024).

### 3.5. Screening of MIC and MBC of PSTO2 and Its Analogues

The essential antimicrobial ability of each peptide and melittin was evaluated using the MIC and MBC assays [21] (Table 3). Based on the results, the MIC of PSTO2 against *S. aureus* and MRSA was 2 μM and 8 μM, while the MBC was 4 μM and 16 μM, which demonstrated prominent anti-Gram-positive bacteria activity. PSTO2 also exhibited inhibitory and bactericidal activity against *E. coli*, with an MIC of 16 μM and an MBC of 32 μM, which indicates it is a broad-spectrum AMP. The analogues of 7K, 18K, SR, and SR2 showed enhanced antimicrobial effects. The analogue of 7K exhibited a two-fold increase in antibacterial activity against MRSA and *E. coli*, a four-fold enhancement against *P. aeruginosa*, and an eight-fold augmentation against *K. pneumoniae*, achieving an MIC of 8 μM. The analogue 18K further improves the antimicrobial activity by four-fold against *E. coli*, *A. baumannii*, *K. pneumoniae*, and *P. aeruginosa*, with MICs of 4 μM, 32 μM, 16 μM, and 32 μM, respectively. As for SR and SR2, these two peptides inhibited and killed most strains at 2 μM and exhibited a more than 32-fold enhancement in antibacterial activity against *A. baumannii*, with MIC values of 4 μM and 2 μM, respectively. Meanwhile, they inhibited the growth of *P. aeruginosa* at a concentration of 16 μM. The data above suggest that the antimicrobial activity of AMPs can be improved with the addition of positive charges, which is consistent with our prediction.

With D-lysine substitution, SRD7 and SR2D10 possessed more potent antibacterial ability against *S. aureus* and MRSA, with an MIC of 1 μM. For Gram-negative bacteria, the antimicrobial activity of the SRD7 against both *A. baumannii* and *K. pneumoniae* exhibited a reduction with MICs of 16 μM and 4 μM compared with SR and SR2. When all the additional lysines were replaced by D-type enantiomers, the antimicrobial effect of Gram-positive bacteria of D1 and D2 disappeared, and its inhibitory effect against *E. coli*, *A. baumannii* and *P. aeruginosa* was retained.

### 3.6. Inhibition and Eradication Ability against Biofilm of PSTO2 and Its Analogues

The inhibitory effect of PSTO2 on biofilm growth and retention of different strains (Table 4) was evaluated with the crystal violet staining method. PSTO2 showed a better inhibitory effect on the growth of *S. aureus* and MRSA biofilms (MBICs = 8 μM and 4 μM) than *E. coli* biofilms (MBIC = 32 μM). Additionally, PSTO2 can eradicate the formed biofilms of *S. aureus* and MRSA at a concentration of 32 μM, while the MBEC against *E. coli* is higher than 128 μM. Analogues of SR and SR2 demonstrated a higher inhibitory effect (MBIC = 2 μM) on the biofilms of Gram-positive bacteria, and their inhibitory ability against *E. coli* biofilms increased by 2–4 fold. This indicates that the lysine substitution of amino acids has an obvious influence on anti-biofilm function. After the modification of D-lysine substitution, the efficacy of SRD7 and SR2D10 against biofilm was diminished compared with SR and SR2.

### 3.7. Salt and Serum Sensitivity

The sensitivity of PSTO2 and its analogues in the presence of salt ions and serum (Table 5) was evaluated, and the MIC and MBC values were tested under different conditions, which are shown in Table 5. The antimicrobial ability of PSTO2 was clearly affected to varying extents against both Gram-positive and Gram-negative bacteria. For *S. aureus*, the MICs of SR, SR2, SRD7, and SRD10 were less influenced in the salt simulation environment and serum conditions, showing lower sensitivity. The MIC of SR against *S. aureus* decreased to 1 μM in the presence of sodium ions, potassium ions, and ferric ions. For *E. coli*, salt ions and serum had more influence on the antimicrobial activity of each peptide.

### 3.8. Time-Killing Kinetics of PSTO2 and Effective Analogues against MRSA and E. coli

**Figure 5:**
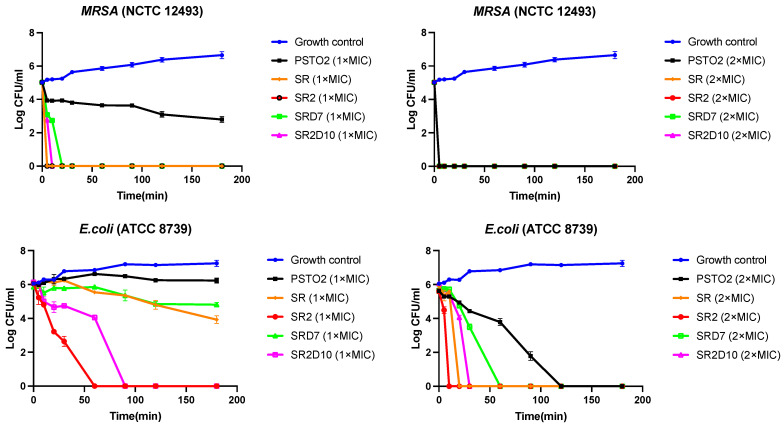
The kinetic time–killing curves of PSTO2 and its analogues against MRSA and *E. coli* at a concentration of 1/2× MIC. Bacteria treated with only a culture medium were used as the negative control. The error bar indicates the SEM of the nine replicates from three repeated tests.

The killing efficiency–time curve was set up with different concentrations to detect the killing rate of PSTO2 and its analogues. As described in Figure 5, PSTO2 inhibited the growth of MRSA at MIC, with more than 1 × 10^3^ CFU/mL of bacteria surviving in 180 min incubation. It instantly killed all the bacteria when the concentration increased to 2× MIC. For *E. coli*, PSTO2 inhibited bacterial growth at 2× MIC after 120 min incubation. The analogues of SR, SR2, SRD7, and SR2D10 generally showed remarkable killing efficiency against MRSA in less than 30 min at 1× MIC and 2× MIC, while SR and SRD7 at MICs reduced the number of viable *E. coli* to around 1 × 10^4^ to 1 × 10^5^ CFU/mL.

### 3.9. Sytox Green Inclusion against Gram-Positive Bacteria by PSTO2 and Selected Analogues

**Figure 6:**
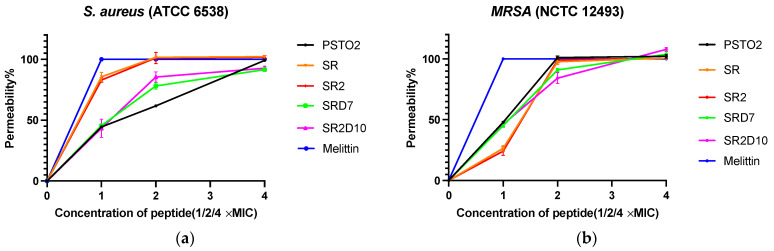
The permeability of bacterial membranes affected by PSTO2 and its selected analogues against (**a**) *S. aureus* and (**b**) MRSA. For the positive control, 16 μM of melittin was used. The error bar indicates the SEM of the nine replicates from three individual tests.

To detect the potential action mechanism between AMPs and Gram-positive bacteria membranes, the Sytox Green staining method was used. Selected peptides in 1×, 2×, and 4× MIC were prepared for comparison with melittin, which was chosen as the positive control (Figure 6). For *S. aureus*, PSTO2 and SRD7 at 1× MIC perturbed about 40% of cells. SR and SR2 caused severe damage to up to 80% of cells, functioning similarly to membrane-penetrating peptides and leading to the leakage of cell contents. When the concentration was raised to 2× or 4× MIC, SR and SR2 disrupted all the cells and contributed to cell death. In the MRSA line chart, the slope of each peptide was lower than that of *S. aureus*, indicating that the permeability of the same peptide differed between strains and that the MRSA membrane was more challenging to penetrate.

### 3.10. NPN Outer Membrane Permeability 

**Figure 7:**
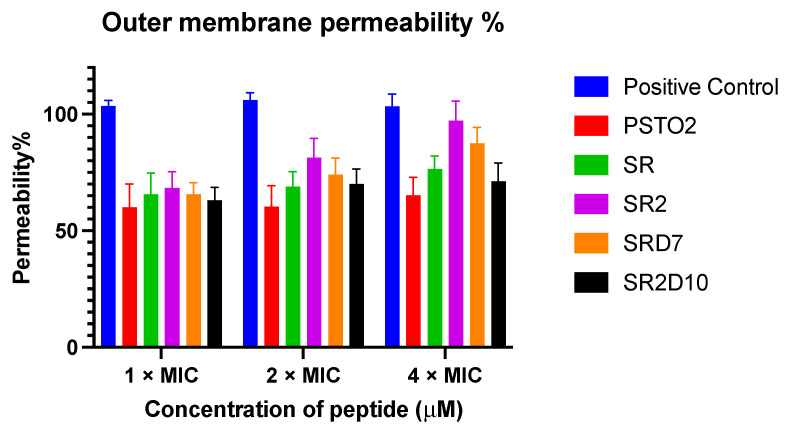
The outer membrane permeability ratio of PSTO2 and its analogues against *E. coli* (ATCC 8739). Bacteria incubated with 16 μM of melittin were used as the positive control. The error bar indicates the SEM of the nine replicates from three repeated tests.

To investigate the penetration of PSTO2 and its analogues on the outer membrane of Gram-negative bacteria, the ability of the peptides to affect the integrity of the outer membrane was quantified by measuring the combination of the NPN fluorescent probe and the outer membrane of *E. coli* (ATCC 8739). This probe is excluded by an intact membrane but binds to the hydrophobic tails of phospholipids when the outer membrane is compromised, resulting in increased fluorescence. Based on the chart (Figure 7), all modifications showed higher outer membrane permeability than PSTO2, and the permeability of each peptide on *E. coli* was concentration-dependent.

### 3.11. LPS Neutralization 

**Figure 8:**
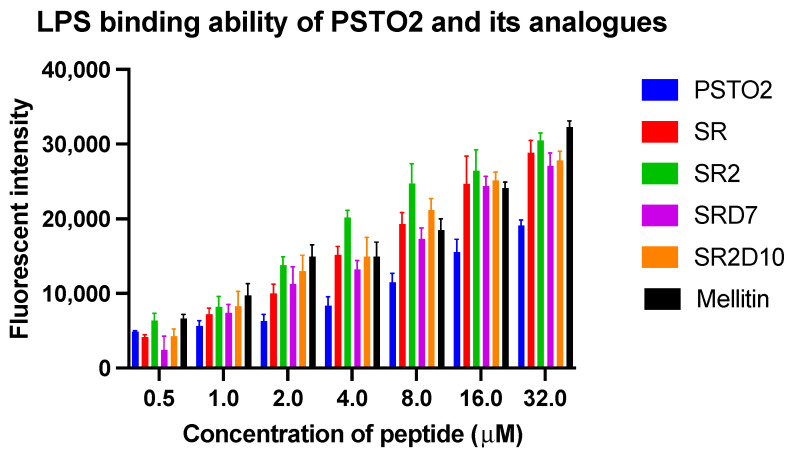
LPS-binding capacity of PSTO2 and its four analogues at different concentrations. Melittin was used as the positive control. The error bar indicates the SEM of the nine replicates from three individual tests.

Considering the outer membrane permeability effect of PSTO2 and its analogues, it is supposed that these peptides can interact with LPS, which is the main component of the outer membrane. To study the combination process of AMPs and bacterial membranes, the LPS-binding ability of peptides was evaluated. Based on the results (Figure 8), each peptide can combine with LPS in a dose-dependent manner, and all the modified peptides exhibited higher binding ability with LPS than PSTO2, which suggested a relationship with the increasing antimicrobial function of peptide analogues against Gram-negative bacteria strains.

### 3.12. In Vivo Antimicrobial Activity in Galleria mellonella Model

**Figure 9:**
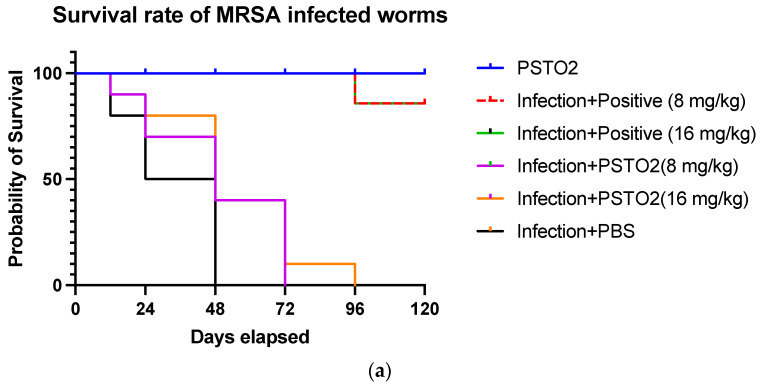

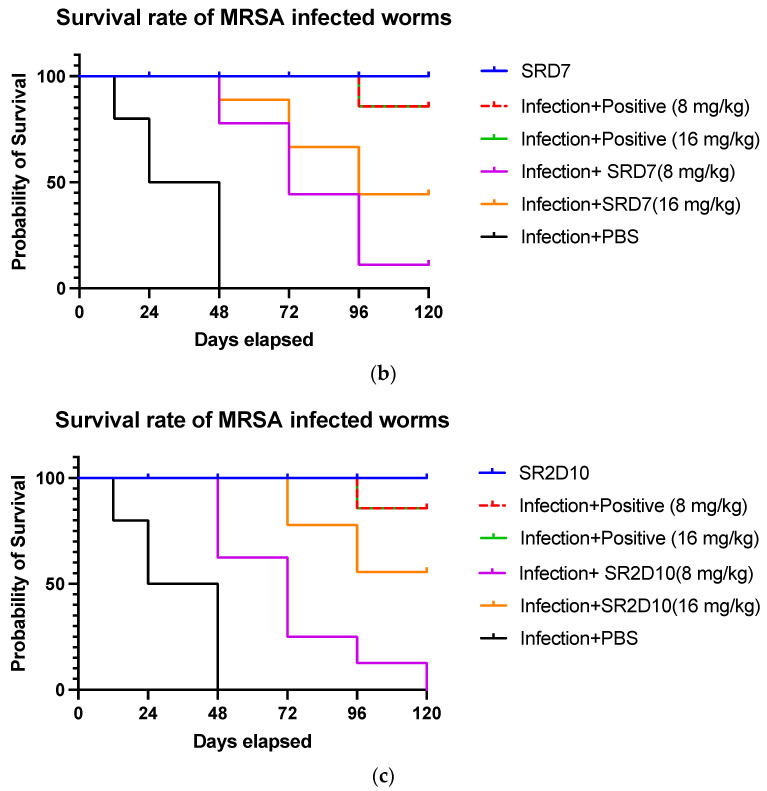
The survival rates of MRSA-infected *Galleria mellonella* larvae after treatment with PSTO2 (**a**), SRD7 (**b**), and SR2D10 (**c**). The infected larvae treated with corresponding vancomycin concentrations were set up as the positive control. The infected larvae treated with PBS were regarded as the negative control.

To evaluate the antimicrobial effect of PSTO2, SRD7, and SR2D10 in vivo, *Galleria mellonella* was chosen to construct infection models. The survival curves (Figure 9) suggest that PSTO2 and its analogues can significantly improve survival in MRSA-infected larvae. At a concentration of 8 mg/kg, SRD7 showed the most potent effect. At a concentration of 16 mg/kg, SRD7 exhibited a 44% survival rate after 120 h treatment, while SR2D10 achieved a 55% survival rate.

### 3.13. Anti-Proliferation Activity against Cancer and Normal Cells

The anti-proliferative ability of PSTO2 and its analogues was assessed on four human cancer cell lines (H838, H460, U251MG, and MCF-7) and two normal cell lines (MRC-5 and HaCaT). The IC_50_ values and SI values were calculated and are shown in Table 6. Based on the data, PSTO2 exhibited an inhibitory effect on the four types of cancer cells and can strongly obliterate 50% of MCF-7 cancer cells at a concentration of 23.10 μM. The four analogues, SR, SR2, SRD7, and SR2D10, showed higher activity in inhibiting the growth of human lung cancer cells in vitro. PSTO2 exhibited varying degrees of inhibitory effects on the four tested cancer cell lines. It was most effective against MCF-7 cells, but PSTO2 had moderate effects on NCI-H460, U251MG, and NCI-H838 cells. According to the selectivity index calculation in Table 7, both the anti-proliferative and cytotoxic effects of SR and SR2, which were designed by increasing the positive charge of the parent peptide, were enhanced. SRD7 and SR2D10 not only demonstrated highly effective inhibition of cancer cells but also exhibited low toxicity, highlighting the selectivity of L-lysine substituted analogues.

### 3.14. Trypan Blue Exclusion Activity

**Figure 10:**
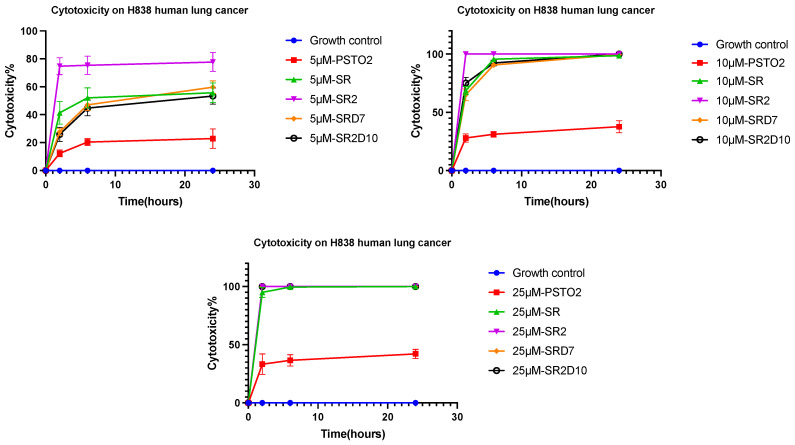
The cytotoxicity of different concentrations of PSTO2 and its analogues expressed by trypan blue targeting H838 human lung cancer cells. Each peptide was prepared separately for 5 μM, 10 μM, and 25 μM for 2, 6, and 24 h. The extent of cytotoxicity was indicated by the ratio of dead cells and total cells. The error bar indicates the SEM of the nine replicates from three individual tests.

To evaluate the cytotoxicity and killing efficiency of PSTO2 and its analogues, a trypan blue exclusion assay was applied against H838 human lung cancer cells. Based on the data shown in Figure 10, the killing efficiency of the PSTO2 analogues increased compared to the parent peptide, and as the concentration of the analogues increased, the speed of killing also increased. All peptides effectively killed around 100% of cells at 10 μM after 24 h incubation, with significantly enhanced fatality at higher concentrations.

### 3.15. Haemolytic Activity 

**Figure 11:**
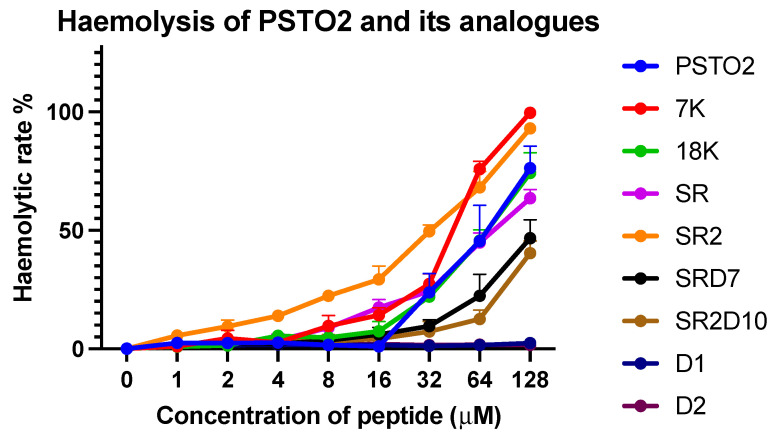
The haemolytic activities of PSTO2 and its analogues at concentrations from 1 to 128 μM. For the positive control, 1% triton X-100 was used, and the haemolytic percentage was calculated based on this. Treatment with PBS was used as the negative control. The error bar indicates the SEM of the nine replicates from three individual tests.

To explore the structure–safety relationship of PSTO2 and its analogues and identify analogues with excellent therapeutic potential and low cytotoxicity, their destructive effect on horse erythrocytes was investigated. Based on Figure 11, analogues with increasing positive charges exhibited more significant haemolysis, and as the concentration of the analogues increased, the haemolysis also increased. However, the haemolysis of D-type lysine substituted analogues was reduced, and D1 and D2 had no effect on horse erythrocytes. Meanwhile, the haemolysis of SRD7 and SR2D10 significantly decreased, and they possessed a similar antimicrobial ability to SR and SR2. The HC_10_ values and therapeutic index of each peptide were calculated for targeting Gram-positive/-negative/yeast (Table 8). There was a significant improvement in the effectiveness evaluation of SRD7 and SR2D10 compared with the original peptide, which indicates that these two analogues showed the most effective therapeutic effects and safety profiles, making them the most promising candidates for antibiotic substitute development.

## 4. Discussion

ESKAPE bacteria, which are highly resistant and virulent, can cause dangerous infections, such as skin and lung inflammation, posing a severe threat to human life [22]. The overuse of traditional antibiotics has led to the development of bacterial resistance, rendering them ineffective against the increasing incidence of hospital-acquired infections. Amphibian AMPs have been proven to have multiple bioactivities, including antimicrobial and anti-cancer activities, which provide potential alternatives to antibiotics that can disrupt the spread of resistant microorganisms. Meanwhile, modification methods are crucial to improve the bioactivities and lower the toxicity of natural peptides, which can accelerate the development of AMPs for clinical use.

In this project, a novel precursor encoding a peptide named PSTO2 was identified from the skin secretion of *Phyllomedusa tomopterna*. Sequence alignment indicated that PSTO2, phylloseptin-PT and phylloseptin-B2 possess conserved sequences in the signal peptide region and acidic spacer region. Also, they contain a highly conserved motif at the N-terminus (FLSLIP-), while the remaining position demonstrates less conservation. This motif can be regarded as the common denominator of the phylloseptin family, but changes in other positions in the sequence could lead to different functional properties for this phylloseptin. PSTO2 has been proven to have primary antimicrobial activity against various microorganisms through MIC/MBC detection. As a broad-spectrum AMP, PSTO2 can effectively inhibit the growth of Gram-positive bacteria. It is hypothesised that the positive charges of PSTO2 can bind to the negatively charged lipids and other molecules present on the surface of bacteria [23]. When AMPs approach the microbial membrane, they undergo a conformational change and adopt an amphipathic α-helical structure. This allows the hydrophobic portion of peptides to insert into the lipid bilayer and leads to the death of the bacteria [24,25]. However, the potency of PSTO2 in combating bacteria was reduced when salt ion solutions and FBS were introduced into the medium. Salt ions can act as a shield, hindering the electrostatic response between the positively charged peptides and the negatively charged bacterial membrane, consequently affecting the MIC/MBC values [26,27].

**Figure 12:**
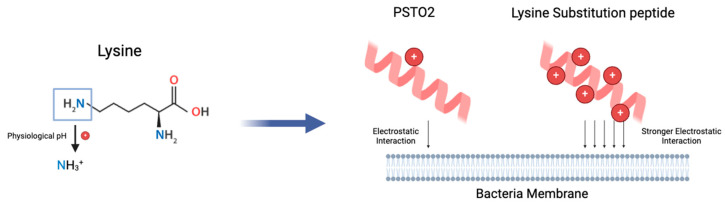
The mode of action of PSTO2 and lysine-substituted peptide with bacteria cell membrane.

Research has proven the importance of positive charge in improving AMP activity by enhancing binding affinity to cell membranes [28,29,30]. Thus, analogues 7K, 18K, SR, and SR2 based on the PSTO2 were designed and exhibited a better inhibitory effect on both Gram-positive and Gram-negative bacteria with lower GM MIC values. As described in Figure 12, with the increase in the number of positive charges donated by lysines, the bactericidal efficiency of the analogues was significantly improved as the membrane interaction increased and can generally cause irreversible death to bacteria within 30 min, according to the time-killing assay. This feature reduces bacterial resistance to AMPs by preventing genetic mutations and quickly stopping the spread of resistance genes. Additionally, analogues with more cations show less susceptibility to the presence of salt ions when combating *S. aureus*, as the positive charges may help them combat salt ions around the bacterial membranes through the ion exchange mechanism, ultimately overcoming the charge shielding effect and increasing their bactericidal effectiveness. Nevertheless, the haemolytic activity of these peptides also increased with the substitution of positively charged amino acids, leading to low TI values, which represents a barrier in AMP development. The utilisation of D-type amino acids has been proven to increase the safety and antimicrobial effect in peptide modification, with a lower possibility of enzyme degradation and haemolysis [31]. Thus, SRD7, SRD10, D1, and D2 were designed by substituting specific amino acids with D-lysines. Interestingly, the antimicrobial effect of SRD7 and SR2D10 against *S. aureus* and MRSA was raised two-fold compared with SR and SR2. This may be due to the ability to fight against proteolytic degradation, which was more challenging to break down, so their antimicrobial activity was improved. In addition, D-amino acids may change the interaction between peptides and bacteria by altering the pore-forming process [32].

PSTO2 and its analogues are more effective in inhibiting Gram-positive bacteria than Gram-negative bacteria. This difference in efficacy can be attributed to the structural differences between these types of bacteria. Gram-negative bacteria have lipopolysaccharides as the main component of their outer membrane, which maintains the stability of their structure, making it more difficult for peptides to penetrate [33,34]. These peptides also showed excellent potential in inhibiting and eradicating biofilms of Gram-positive bacteria at a low concentration. This capability offers promise for treating biofilm infections common in clinical settings, such as catheter infections. Biofilms are more challenging to inhibit than planktonic bacteria. This could be attributed to the complex structure of biofilms, which are formed by microbial communities encased in a matrix of extracellular polymeric substances (EPSs). Such a structure limits the diffusion of AMPs into their inner layers. Additionally, microbial cells in biofilms may develop adaptive resistance mechanisms that decrease peptide effectiveness by triggering changes in gene expression and physiological responses [35,36]. According to the results, the inhibitory and eradication effects of these peptides on biofilms increase with the increment of positive charges, and the change in conformation on these peptides has little impact on their anti-biofilm activity, and relevant studies have demonstrated that D-type amino acids also possess the capability to combat biofilms [37,38].

Further exploration of the antibacterial action mechanisms reveals that AMPs generally interact with phospholipid membranes through various mechanisms, leading to membrane damage and intracellular content leakage. This can be summarised using barrel-stave, toroidal pore, or carpet models [39,40].

In the Sytox Green permeability assay, PSTO2 and its analogues can disrupt the integrity of the cell membrane of *S. aureus* and MRSA, and the process is related to the concentration and positive charge number of peptides. With the highest cation number, SR2 demonstrated the strongest impact on the membrane, reached 100% membrane permeability rate against the two strains at 2× MIC and 4× MIC, and led to cell membrane rupture and bacterial death, which suggests the crucial role of electrostatic adsorption in the action of AMPs. 

The outer membrane plays a crucial role in Gram-negative bacteria as a selective osmotic barrier, which can prevent the entry of some biomacromolecules and is generally more difficult to kill or inhibit [41]. Meanwhile, LPS is involved in constructing cell membrane integrity and can induce immune responses to protect bacteria from invasion. In the NPN uptake test, PSTO2 had a relatively weak destructive effect on the outer membrane compared with the four tested analogues. As the number of positive charges increased, the permeability of these AMPs on the outer membrane of Gram-negative bacteria progressively increased, thereby contributing to their antimicrobial activity against these bacteria. Notably, SR2 reached a permeability level approaching 100%, comparable to that induced by melittin at 4× MIC, suggesting a potentially similar mechanism of action of melittin. Furthermore, compared to PSTO2, the analogues showed a significantly enhanced binding affinity to LPS components, indicating the impact of AMPs on the outer membrane of Gram-negative bacteria and highlighting the importance of adding positive charges to improve membrane-binding efficacy.

Considering the similar membrane characteristics, the anti-cancer activity of AMPs is also meaningful to explore. Several AMPs with anti-cancer abilities were reported in recent research [42,43,44]. Based on the results of MTT assays, PSTO2 was effective in inhibiting the growth of various cancer cells, including H838, H460, MCF-7, and U251MG after 24 h peptide treatment. After modification, SR and SR2 had the most substantial anti-cancer effects, and SRD7 and SR2D10 demonstrated notable effectiveness specifically against human lung cancer, which provides potential in cancer therapy research. In the trypan blue assays, PSTO2 and its analogues exhibited complete cytotoxicity against lung cancer within 24 h, demonstrating a rapid lethal effect. Simultaneously, the distinct activity difference of SRD7 and SR2D10 between cancerous and normal cells lays the foundation for the safety of AMPs as anti-cancer drugs. Similar to antimicrobial mechanisms, the positively charged AMPs may have a higher affinity to the negatively charged phospholipids on the cancer cell membrane, and the recognition of these components may contribute to the selectivity of AMPs. Another potential mechanism may involve the induction of apoptosis. This is characterised by the promotion of cytochrome C release and activation of the caspase pathway, ultimately leading to programmed cell death [45,46].

Finally, the haemolysis and toxic effects of PSTO2 and its analogues were tested. Regarding the result of the survival assay in vivo, PSTO2 could hardly rescue the waxworms with MRSA infection, while SRD7 and SR2D10 could significantly reduce the mortality of waxworms at safe concentrations. Nearly 50% of the samples could be healed with 16 mg/kg of treatment. It could be concluded that adding D-type amino acids can improve the stability and bioavailability of AMPs, and it also shows the safety of two D-lysine substitutions, which suggests development potential in clinical studies. In haemolysis experiments, an increase in charge led to enhanced haemolytic activity. Compared with PSTO2, the haemolytic activity of 7K, 18K, SR, and SR2 was gradually enhanced as the positive charge number increased. The incorporation of D-lysines significantly reduced the haemolysis of products SRD7 and SR2D10, thereby increasing their therapeutic index. Upon analysis, SRD7 exhibits the strongest research potential in the field of antimicrobial agents.

## 5. Conclusions

In conclusion, PSTO2 was successfully modified by adding positive charges and substituting certain amino acids with D-lysine, which improved the activity and safety of the parent peptide. The D-type modifications, SRD7 and SR2D10, could effectively kill the antibiotic-resistant bacteria through membrane disruption and inhibit the growth of human lung cancer cells without resulting in severe cytotoxicity. Therefore, D-lysine can play a vital role in AMP design, and these two analogues also have desired prospects for development as antibiotic alternatives and anti-cancer drugs.

## Figures and Tables

**Table 1:** Peptide sequences and the physicochemical properties of PSTO2 and its analogues.

Peptide	Sequence	Net Charge	Hydrophobicity
PSTO2	FLSLIPHAISAVSALAKHL-NH_2_	1	0.764
PSTO2-7K	FLSLIPKAISAVSALAKHL-NH_2_	2	0.705
PSTO2-18K	FLSLIPKAISAVSALAKKL-NH_2_	3	0.646
PSTO2-SR	FLSLIPKAIKAVSALAKKL-NH_2_	4	0.596
PSTO2-SR2	FLSLIPKAIKAVKALAKKL-NH_2_	5	0.546
PSTO2-D1	FLSLIPKdAIKdAVSALAKKdL-NH_2_	4	0.596
PSTO2-D2	FLSLIPKdAIKdAVKdALAKdKdL-NH_2_	5	0.546
PSTO2-SRD7	FLSLIPKdAIKAVSALAKKL-NH_2_	4	0.596
PSTO2-SR2D10	FLSLIPKdAIKAVKALAKKdL-NH_2_	5	0.546

**Table 2:** The helical percentage prediction of PSTO2 and its analogues in 50% TFE/10 mM of NH_4_A_C_ and 10 mM of NH_4_A_C_.

Peptide	50% TFE/10 mM of NH_4_Ac			10 mM of NH_4_Ac		
	α Helix (%)	β Strand (%)	Others (%)	α Helix (%)	β Strand (%)	Others (%)
PSTO2	95.36	0.02	4.62	1.86	8.93	89.21
7K	95.14	0.02	4.84	13.82	6.42	79.76
18K	95.36	0.02	4.62	1.84	8.94	89.22
SR	95.09	0.02	4.89	9.19	6.83	83.98
SR2	95.10	0.02	4.88	2.81	8.41	88.78
SRD7	94.34	0.01	5.65	1.82	9.24	88.94
SR2D10	83.88	0.04	16.08	1.80	9.21	88.99
D1	66.21	0.20	33.59	1.77	9.22	89.01
D2	54.71	0.90	44.39	1.26	10.21	88.53

**Table 3:** MICs and MBCs of PSTO2 and its analogues against different microorganisms.

		PSTO2	7K	18K	SR	SR2	SRD7	SR2D10	D1	D2	Mellitin
	**MIC/MBC (μM)**										
Gram-positive	*S. aureus* (ATCC 6538)	2/4	4/4	2/4	2/2	2/2	1/1	1/1	>128	>128	2/2
	MRSA (NCTC 12493)	8/16	4/8	4/4	2/2	2/2	1/2	1/1	>128	>128	2/2
Gram-negative	*E. coli* (ATCC 8739)	16/32	8/8	4/4	2/2	2/2	2/2	2/2	32/32	16/32	4/4
	*A. baumannii* (BAA 747)	>128	>128	32/32	4/4	2/2	16/16	16/16	32/32	16/16	NA
	*K. pneumonia* (ATCC 43816)	64/128	8/8	16/16	2/2	2/2	4/4	4/4	128/128	128/128	32/32
	*P. aeruginosa* (ATCC 9027)	>128	32/32	32/64	16/32	16/32	16/16	32/32	32/32	16/16	16/32
Yeast	*C. albicans*	32/128	32/64	32/128	32/128	32/64	32/64	64/128	>128	>128	NA
	**MIC/MBC (μg/mL)**										
Gram-positive	*S. aureus* (ATCC 6538)	4.0/8.0	7.9/7.9	3.9/7.9	4.0/4.0	4.1/4.1	2.0/2.0	2.1/2.1	>257.3	>262.5	5.7/5.7
	MRSA (NCTC 12493)	15.9/31.8	7.9/15.8	7.9/7.9	4.0/4.0	4.1/4.1	2.0/4.0	2.1/2.1	>257.3	>262.5	5.7/5.7
Gram-negative	*E. coli* (ATCC 8739)	31.8/63.6	15.8/15.8	7.9/7.9	4.0/4.0	4.1/4.1	4.0/4.0	4.1/4.1	64.3/64.3	32.8/65.6	11.4/11.4
	*A. baumannii* (BAA 747)	>254.3	>253.2	63/63	8.0/8.0	4.1/4.1	32.2/32.2	32.8/32.8	64.3/64.3	32.8/32.8	NA
	*K. pneumonia* (ATCC 43816)	127.2/254.3	15.8/15.8	31.5/31.5	4.0/4.0	4.1/4.1	8.0/48.0	8.2/48.2	257.3/257.3	262.5/262.5	91.1/91.1
	*P. aeruginosa* (ATCC 9027)	>254.3	63.3/63.3	63/126	32.2/64.3	32.8/65.6	32.2/32.2	65.6/65.6	64.3/64.3	32.8/32.8	45.5/91.1
Yeast	*C. albicans*	63.9/254.3	63.3/126.6	63/252.1	64.3/257.3	65.6/131.3	64.3/128.7	131.3/262.5	>257.3	>262.5	NA

NA means not applicable.

**Table 4:** MBIC and MBEC of PSTO2 and its analogues against different bacteria.

		PSTO2	7K	18K	SR	SR2	SRD7	SR2D10
MBIC (μM)/MBEC (μM)								
Gram-positive	*S. aureus* (ATCC 6538)	8/32	16/32	8/32	2/16	2/16	2/32	4/32
	MRSA (ATCC 12493)	4/32	4/32	4/32	2/16	2/16	4/16	16/32
Gram-negative	*E. coli* (ATCC 8739)	32/>128	16/64	32/64	16/16	8/16	32/64	32/64

**Table 5:** The MICs of PSTO2 and its analogues against *S. aureus* and *E. coli* in diverse salts.

***S. aureus*** **(ATCC 6538)**								
**MIC (μM)/MBC (μM)**								
**Peptide**	**Medium**	**NaCl**	**KCl**	**NH_4_Cl**	**CaCl_2_**	**MgCl_2_**	**FeCl_3_**	**10% FBS**
PSTO2	2/4	8/8	16/16	64/64	16/32	16/64	16/16	8/16
SR	2/2	1/1	1/1	2/2	2/4	2/2	1/2	2/2
SR2	2/2	2/2	2/2	2/2	2/2	2/2	2/2	2/2
SRD7	1/1	4/4	2/2	2/2	2/2	2/2	2/2	2/2
SR2D10	1/1	4/4	2/2	2/2	2/2	2/2	2/2	2/2
***E. coli*** **(ATCC 8739)**								
**MIC (μM)/MBC (μM)**								
**Peptide**	**Medium**	**NaCl**	**KCl**	**NH_4_Cl**	**CaCl_2_**	**MgCl_2_**	**FeCl_3_**	**10% FBS**
PSTO2	16/32	64/128	32/64	32/128	>128>128	32/64	16/16	>128
SR	2/2	16/32	4/4	2/4	16/32	2/4	2/2	8/16
SR2	2/2	4/4	4/4	4/4	8/16	4/4	2/2	8/16
SRD7	2/2	8/8	4/4	2/4	64/64	8/8	2/2	8/8
SR2D10	2/2	4/4	4/4	2/4	32/32	4/4	2/2	8/8

**Table 6:** The IC_50_ values of PSTO2 and its analogues against various cancer and normal cell lines.

IC_50_ (μM)						
	NCI-H838	NCI-H460	U251MG	MCF-7	MRC-5	HaCat
PSTO2	85.79	78.28	84.03	23.10	73.22	70.58
SR	2.88	3.07	9.65	6.05	6.98	4.13
SR2	3.13	5.92	6.72	7.83	6.56	3.48
SRD7	3.32	2.31	13.86	15.18	77.28	77.89
SR2D10	9.49	7.70	16.05	26.38	74.39	84.03

**Table 7:** The selective index (SI) under IC_50_ of PSTO2 and its effective analogues.

	PSTO2	SR	SR2	SRD7	SR2D10
SI_(MRC-5)_/SI_(HaCat)_ *					
H838	0.85/0.82	2.39/1.43	2.09/1.11	23.28/23.46	7.84/8.85
H460	0.94/0.90	2.27/1.35	1.11/0.59	33.45/33.72	9.66/10.91
U251MG	0.87/0.84	0.72/0.43	0.98/0.52	5.58/5.62	4.63/5.24
MCF-7	3.17/3.06	1.15/0.68	0.84/0.44	5.09/5.13	2.82/3.19

* Calculation formula of SI: SI = IC_50(MRC-5/HaCat)_/IC_50(Cancer cell)._

**Table 8:** The HC_10_ values and therapeutic index (TI) under HC_10_ of PSTO2 and its analogues.

Peptide	HC_10_	GM MIC (G+/G−/Yeast)	TI Value under HC_10_
PSTO2	21.78	4/128/32	5.45/0.17/0.68
7K	21.97	4/32/32	5.49/0.69/0.69
18K	20.59	2.8/16/32	7.35/1.29/0.64
SR	12.60	2/4/32	6.3/3.15/0.39
SR2	6.53	2/3.4/32	3.27/1.92/0.2
SRD7	74.78	1/6.7/32	74.78/11.16/2.34
SR2D10	75.24	1/8/64	75.24/9.41/1.18
D1	Not significant	512/45.3/512	N/A
D2	Not significant	512/27/512	N/A

## Data Availability

The phylloseptin-TO2 (PSTO2) biosynthetic precursor encoding cDNA was deposited in the NCBI database (accession No. PP858895).

## Supplementary Materials

The following supporting information can be downloaded at: https://www.mdpi.com/article/10.3390/pharmaceutics16081098/s1, Figure S1: RP-HPLC chromatograms of purified peptides, (a) PSTO2, (b) 7K, (c) 18K, (d) SR, (e) SR2, (f) SRD7, (g) SR2D10, (h) D1, and (i) D2; Figure S2: MALDI-TOF spectra of pure (a) PSTO2 and its analogues, (b) 7K, (c) 18K, (d) SR, (e) SR2, (f) SRD7, (g) SR2D10, (h) D1, and (i) D2.
