# Supplementary material for: Enhancement of Antimicrobial Function by L/D-Lysine Substitution on a Novel Broad-Spectrum Antimicrobial Peptide, Phylloseptin-TO2: A Structure-Related Activity Research Study
Source: Pharmaceutics. 2024 Aug 21;16(8):1098. doi: 10.3390/pharmaceutics16081098 (PMC11360180; doi:10.3390/pharmaceutics16081098)
Supplement: Supplementary file 1 [file pharmaceutics-16-01098-s001.zip › pharmaceutics-3060509-SI.pdf]

Supplementary materials

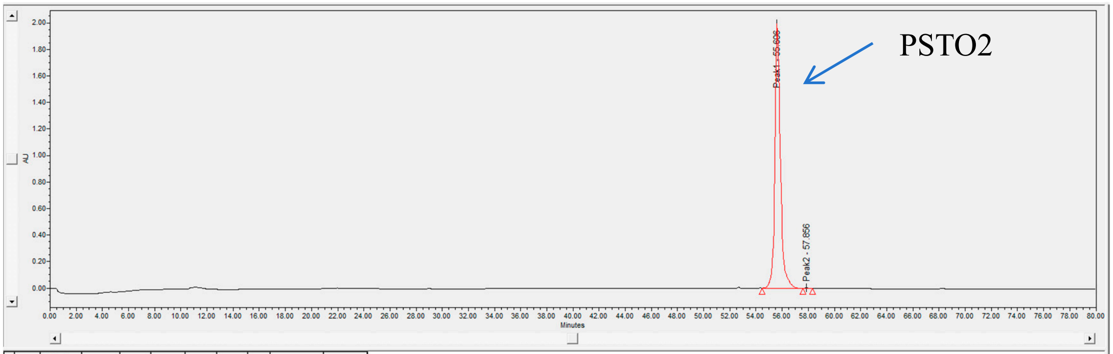

	Name	Retention Time (min)	Area (µV*sec)	% Area	Height (µV)	Int Type	Amount	Units	Peak Type	Peak Codes
1	Peak1	55.606	58289667	99.81	1991645	BB			Found	Q20
2	Peak2	57.856	111557	0.19	8330	BB			Found	Q20

(a)

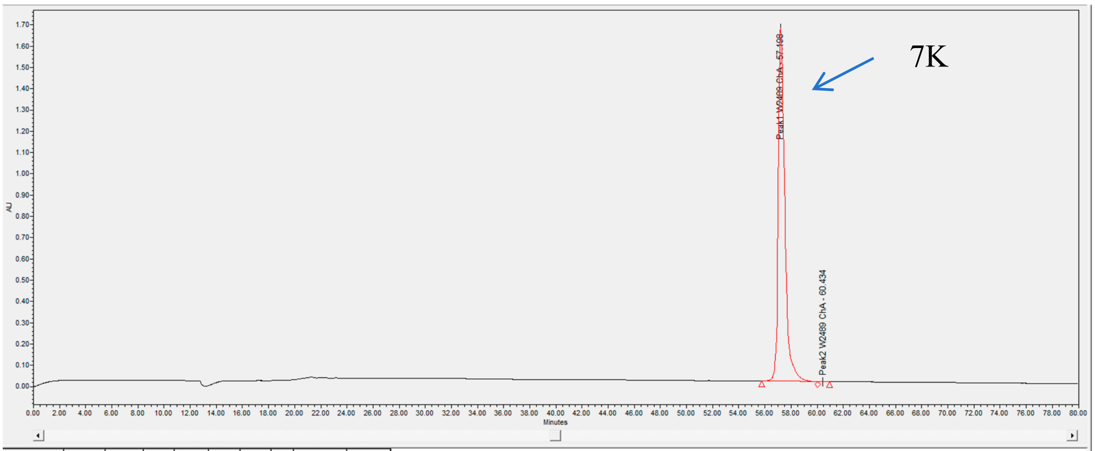

Name	Retention Time (min)	Area (µV*sec)	% Area	Height (µV)	Int Type	Amount	Units	Peak Type	Peak Codes
Peak1 W2489 ChA	57.198	57943376	99.99	1658720	BV			Found	Q20
Peak2 W2489 ChA	60.434	7967	0.01	337	VB			Found	Q20

(b)

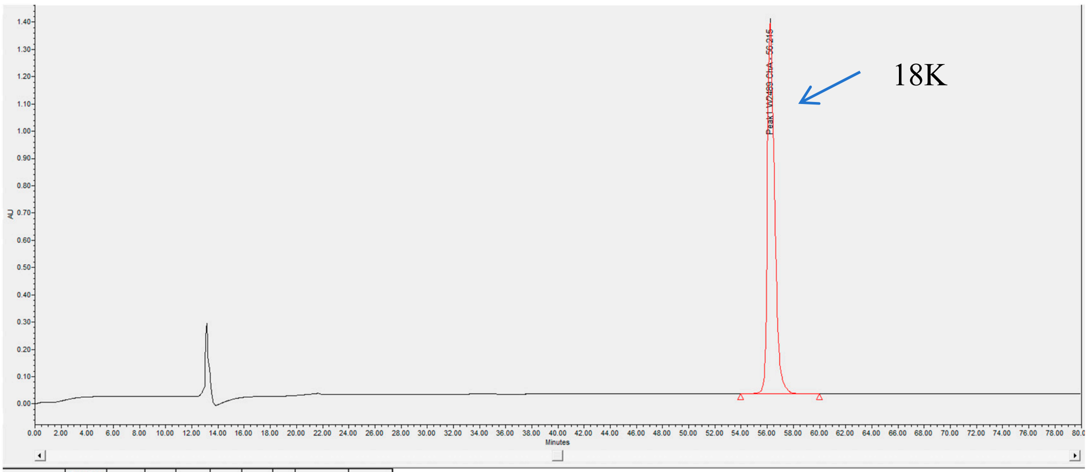

Name	Retention Time (min)	Area (µV*sec)	% Area	Height (µV)	Int Type	Amount	Units	Peak Type	Peak Codes
Peak1 W2489 ChA	56.215	52708984	100.00	1358300	BB			Found	Q20

Activate Windows

(c)

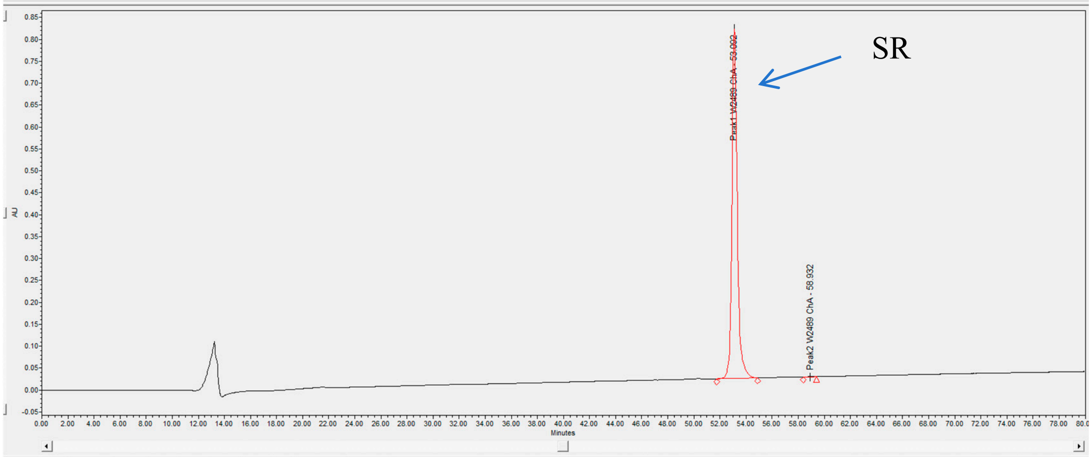

Name	Retention Time (min)	Area (µV*sec)	% Area	Height (µV)	Int Type	Amount	Units	Peak Type	Peak Codes
Peak1 W2489 ChA	53.092	22802848	99.95	796756	VV			Found	Q20
Peak2 W2489 ChA	58.932	11491	0.05	506	VB			Found	Q20

(d)

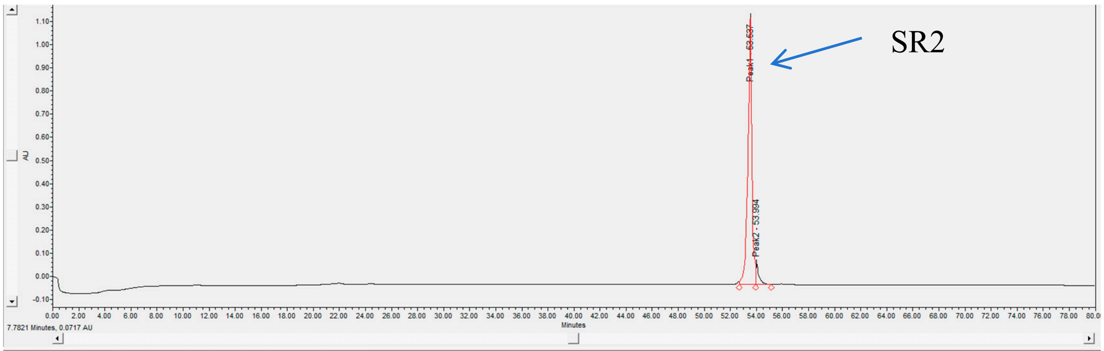

E	Name	Retention Time (min)	Area (µV*sec)	% Area	Height (µV)	Int Type	Amount	Units	Peak Type	Peak Codes
1	Peak1	53.537	24531058	94.22	1148247	VV			Found	Q20
2	Peak2	53.994	1503981	5.78	91311	VV			Found	Q20

(e)

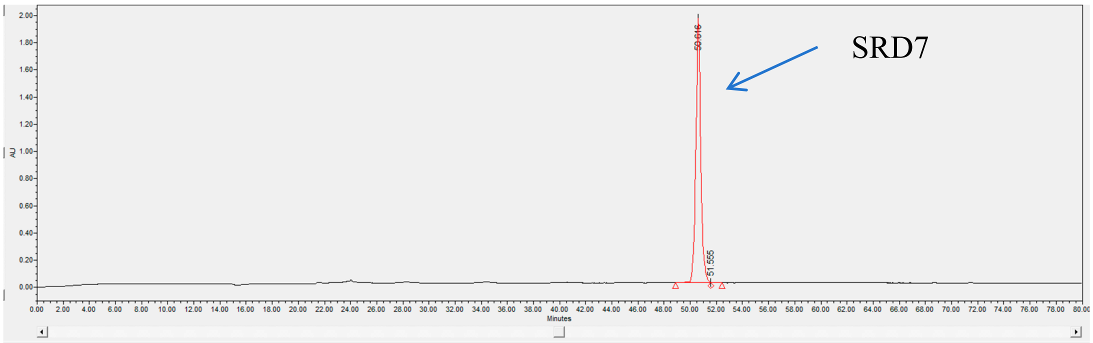

Name	Retention Time (min)	Area (µV*sec)	% Area	Height (µV)	Int Type	Amount	Units	Peak Type	Peak Codes
	50.616	50219579	99.90	1945250	Bv			Unknown	I37
	51.555	47983	0.10	5385	vB			Unknown	I37 I06 I21 I22 I23

(f)

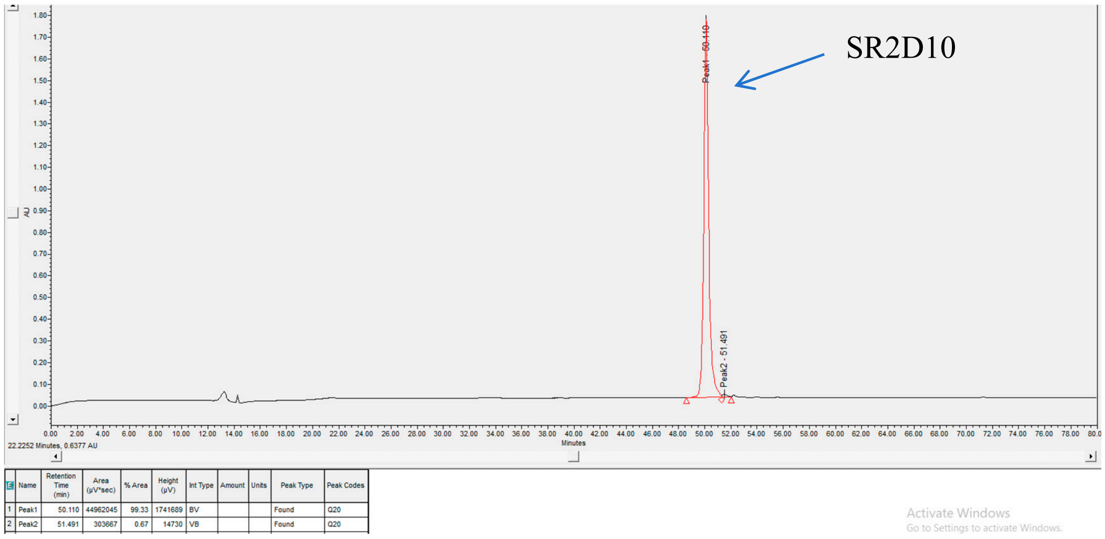

E	Name	Retention Time (min)	Area (μV*sec)	% Area	Height (μV)	Int Type	Amount	Units	Peak Type	Peak Codes
1	Peak1	50.110	44962045	99.33	1741689	BV			Found	Q20
2	Peak2	51.491	303667	0.67	14730	VB			Found	Q20

Activate Windows
Go to Settings to activate Windows.

(g)

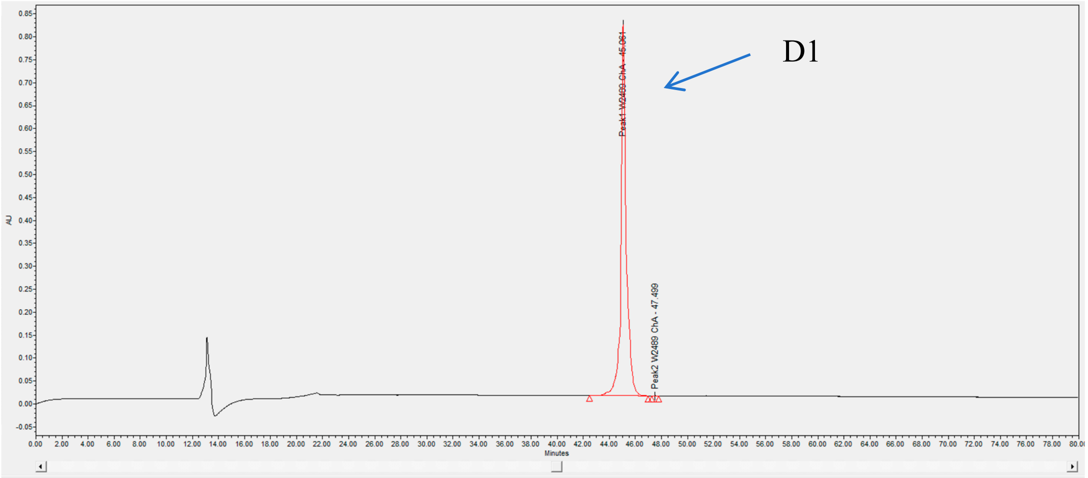

Name	Retention Time (min)	Area (μV*sec)	% Area	Height (μV)	Int Type	Amount	Units	Peak Type	Peak Codes
Peak1 W2489 ChA	45.061	22729538	99.99	808125	BB			Found	Q20
Peak2 W2489 ChA	47.499	1661	0.01	109	BB			Found	Q20

Activate Windows
Go to Settings to activate Windows.

(h)

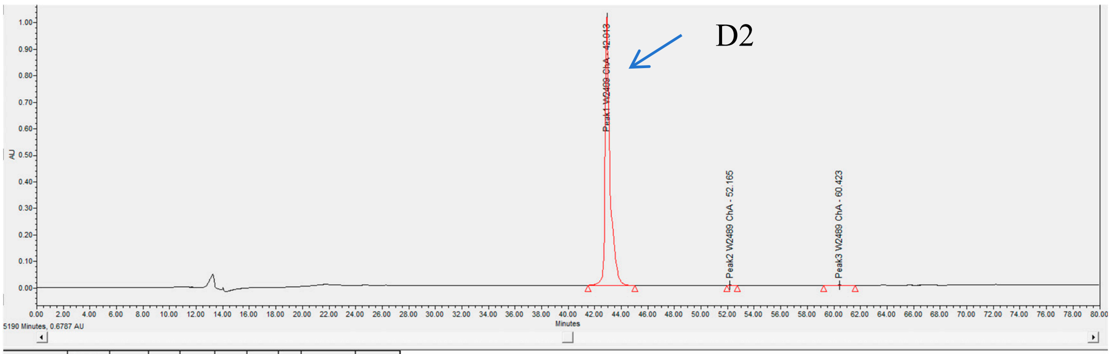

Name	Retention Time (min)	Area (μV*sec)	% Area	Height (μV)	Int Type	Amount	Units	Peak Type	Peak Codes
Peak1 W2489 ChA	42.913	25962535	99.77	1010734	BB			Found	Q20
Peak2 W2489 ChA	52.165	25210	0.10	1553	BB			Found	Q20
Peak3 W2489 ChA	60.423	35856	0.14	1340	BB			Found	Q20

(i)

Figure S1. RP-HPLC chromatograms of purified peptides: (**a**) PSTO2, (**b**) 7K, (**c**) 18K, (**d**) SR, (**e**) SR2, (**f**) SRD7, (**g**) SR2D10, (**h**) D1, and (**i**) D2.

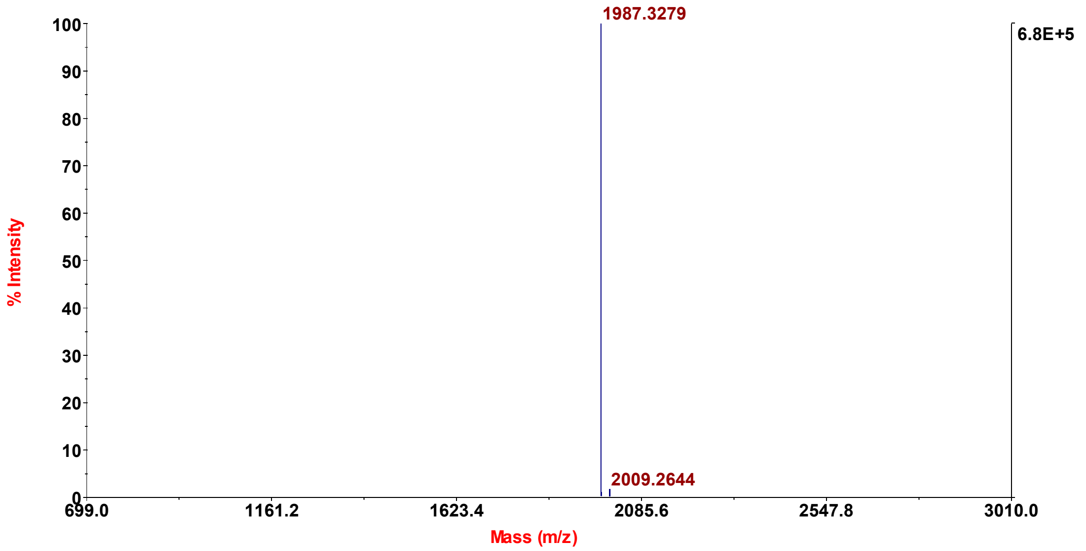
1987.3279
6.8E+5
2009.2644
% Intensity
Mass (m/z)
699.0
1161.2
1623.4
2085.6
2547.8
3010.0

(a)

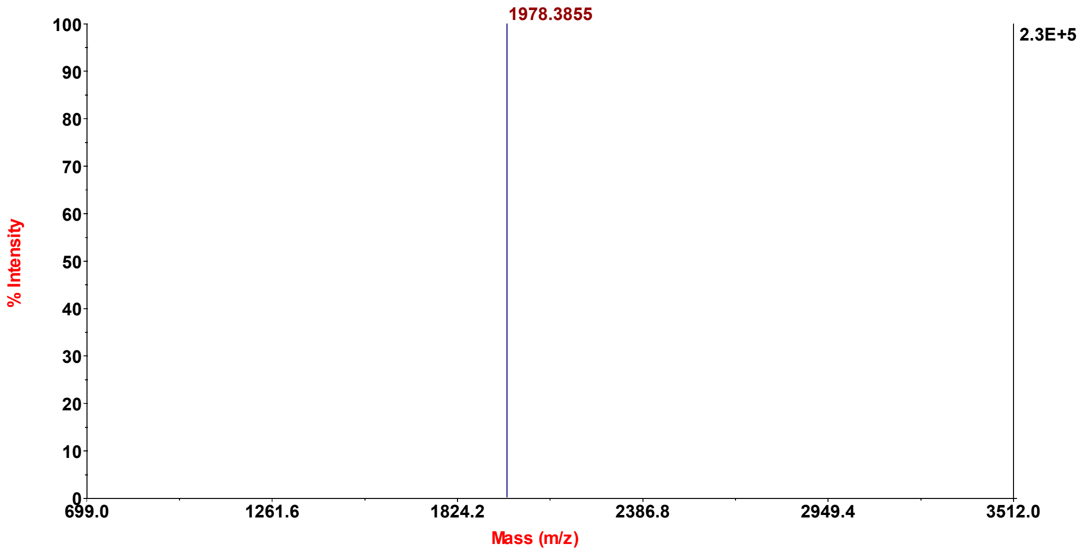
1978.3855
2.3E+5
% Intensity
Mass (m/z)
699.0
1261.6
1824.2
2386.8
2949.4
3512.0

(b)

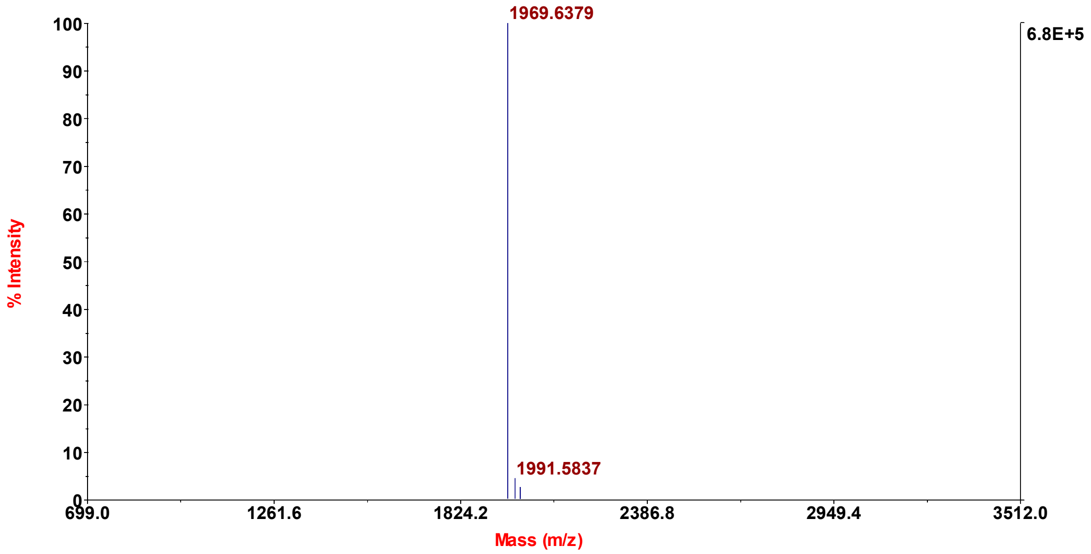
1969.6379
6.8E+5
1991.5837
% Intensity
Mass (m/z)
699.0
1261.6
1824.2
2386.8
2949.4
3512.0

(c)

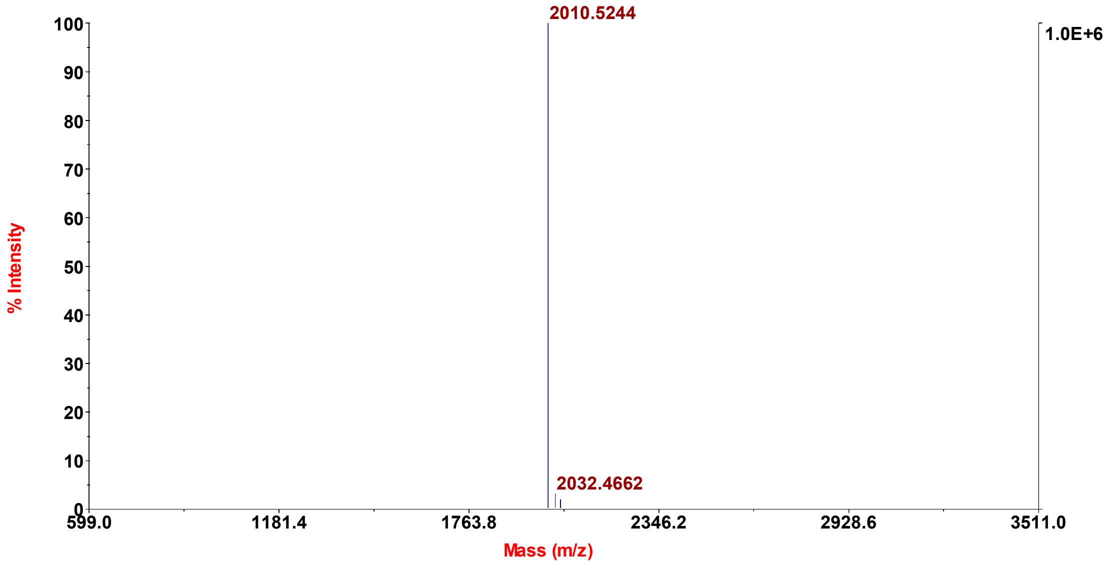
2010.5244
2032.4662
1.0E+6
% Intensity
100
90
80
70
60
50
40
30
20
10
0
599.0
1181.4
1763.8
2346.2
2928.6
3511.0
Mass (m/z)

(d)

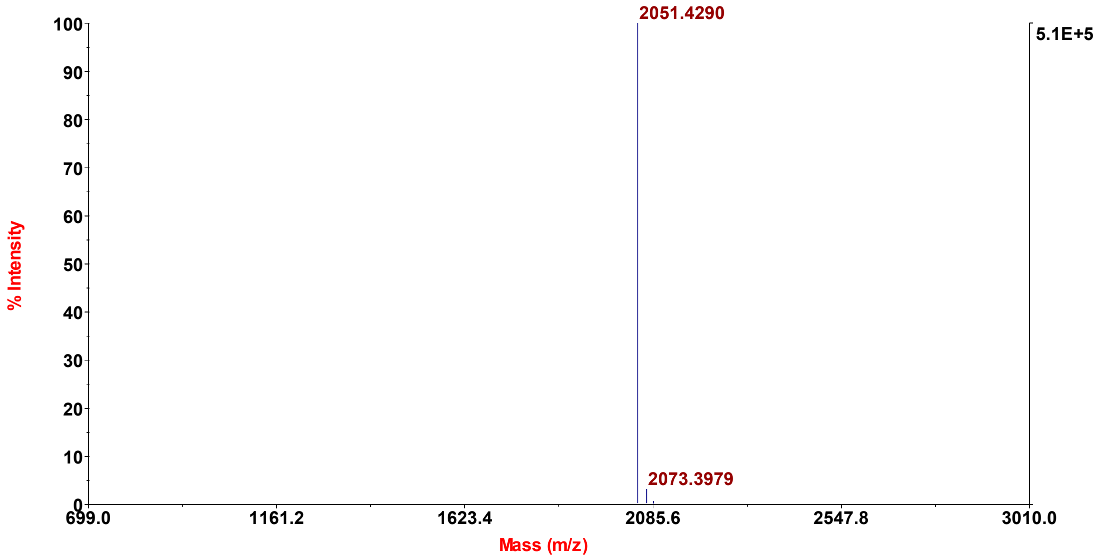
2051.4290
2073.3979
5.1E+5
% Intensity
100
90
80
70
60
50
40
30
20
10
0
699.0
1161.2
1623.4
2085.6
2547.8
3010.0
Mass (m/z)

(e)

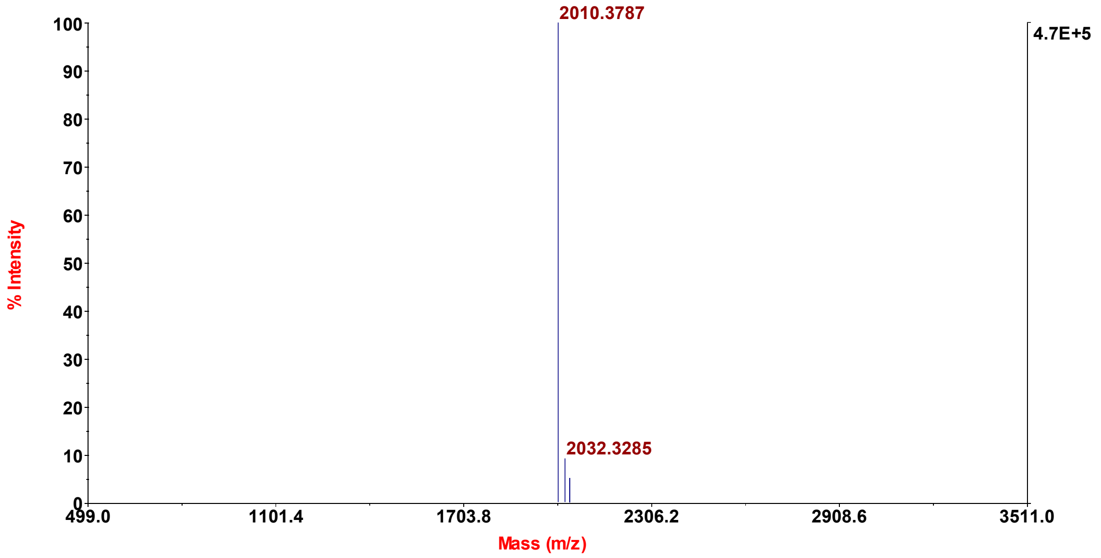
2010.3787
2032.3285
4.7E+5
% Intensity
100
90
80
70
60
50
40
30
20
10
0
499.0
1101.4
1703.8
2306.2
2908.6
3511.0
Mass (m/z)

(f)

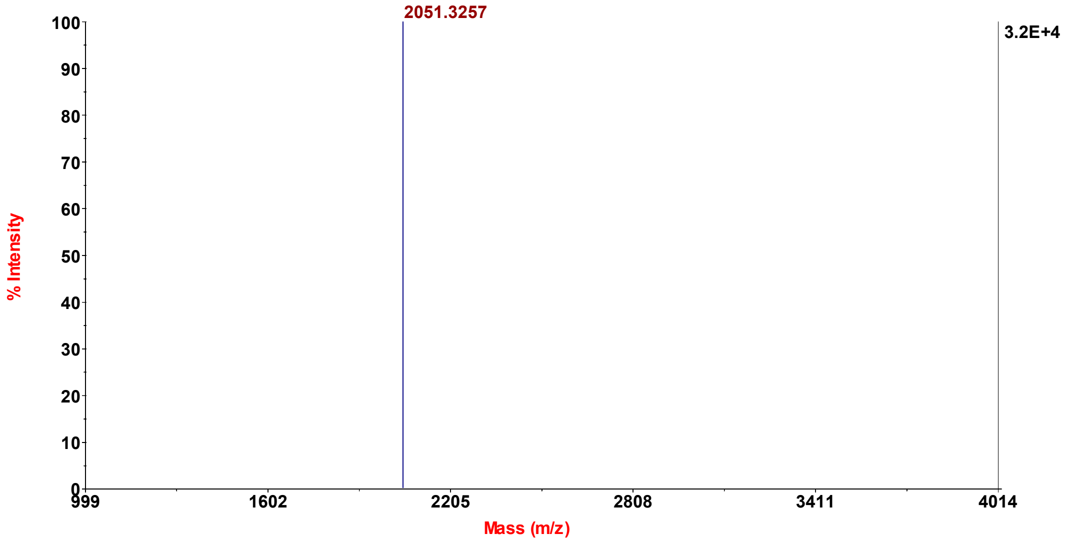

(g)

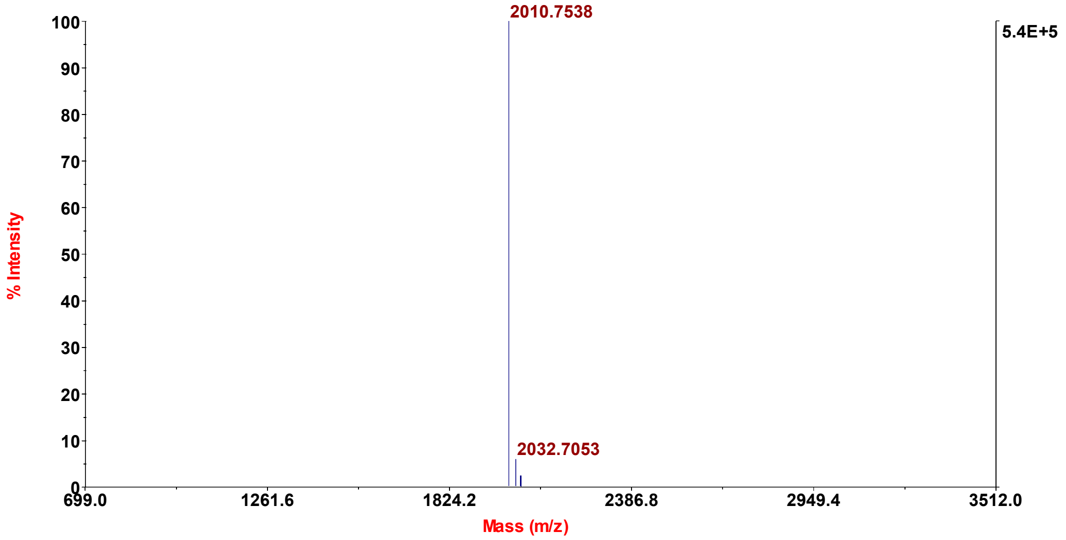

(h)

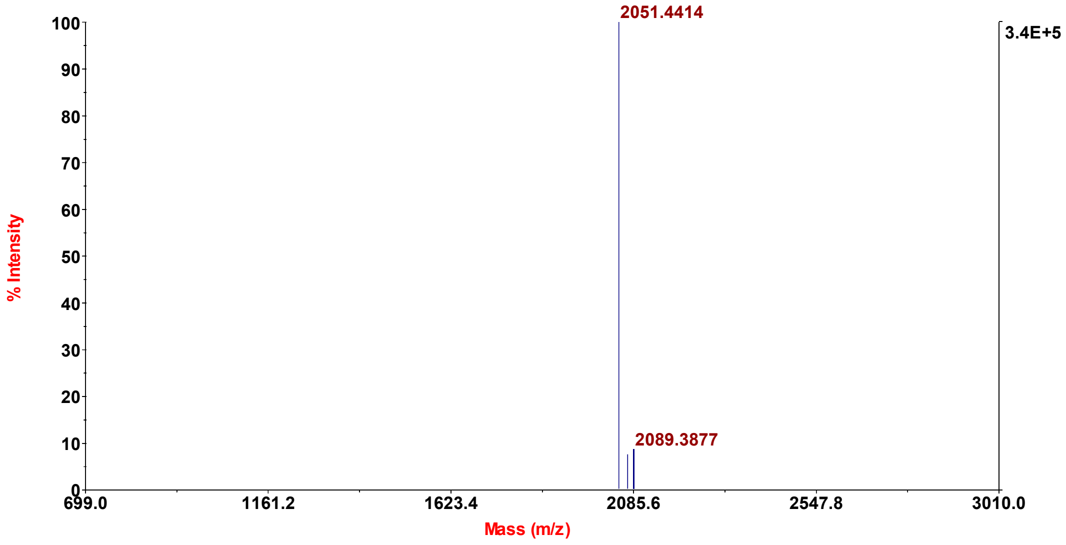

(i)

Figure S2. MALDI-TOF spectra of pure (**a**) PSTO2 and its analogues, (**b**) 7K, (**c**) 18K, (**d**) SR, (**e**) SR2, (**f**) SRD7, (**g**) SR2D10, (**h**) D1, and (**i**) D2.
